# Mechanisms of Aristolochic Acid Resistance in Specialist Butterflies and Evolutionary Insights for Potential Protective Pathways

**DOI:** 10.1002/advs.202518072

**Published:** 2026-01-04

**Authors:** Yang Luan, Yubo Zhang, Jingjing Li, Jianqing Zhu, Yuyang Lei, Yushi Hu, Zhenqiang Xin, Tianpei Xie, Jiang Zheng, Yuanyuan Lin, Jingjing Shen, Yiyi Cao, Xinyue You, Jing Xi, Jiaying Wu, Weiying Liu, Xinyu Zhang, Yuanting Zheng, David J. Lohman, Leming Shi, Wei Zhang

**Affiliations:** ^1^ School of Public Health Shanghai Jiao Tong University School of Medicine Shanghai China; ^2^ State Key Laboratory of Gene Function and Modulation Research School of Life Sciences Peking University Beijing China; ^3^ State Key Laboratory of Genetic Engineering School of Life Sciences and Human Phenome Institute Fudan University Shanghai China; ^4^ Shanghai Zoological Park Shanghai China; ^5^ Shanghai Standard Technology Co., Ltd Shanghai China; ^6^ Wuya College of Innovation Shenyang Pharmaceutical University Shenyang China; ^7^ Laboratory of Discovery and Utilization of Functional Components in Traditional Chinese Medicine, Engineering Research Center for the Development and Application of Ethnic Medicine and TCM (Ministry of Education), Guizhou Provincial Engineering Research Center for the Development and Application of Ethnic Medicine and TCM Guizhou Medical University Guiyang China; ^8^ Shimazu (China) Co., Ltd Shanghai Branch China; ^9^ School of Public Health Chengdu University of Traditional Chinese Medicine Chengdu China; ^10^ Department of Biology City College of New York New York New York USA; ^11^ Ph.D. Program in Biology Graduate Center City University of New York New York USA; ^12^ Entomology Section National Museum of Natural History Manila Philippines; ^13^ Peking‐Tsinghua Center for Life Sciences Academy for Advanced Interdisciplinary Studies Peking University Beijing China; ^14^ Peking University Chengdu Academy for Advanced Interdisciplinary Biotechnologies Chengdu China; ^15^ Medog Biodiversity Observation and Research Station of Xizang Autonomous Region Nyingchi China; ^16^ Institute of Ecology Peking University Beijing China

**Keywords:** antioxidant, Aristolochiaceae, butterflies, chemical defense, DNA repair, multiomics

## Abstract

Aristolochic acids (AAs) are natural compounds found in Aristolochiaceae plants, to which humans are frequently exposed through environmental and medicinal sources. AAs are highly nephrotoxic and carcinogenic, mediated by oxidative stress and bioactivation‐induced DNA damage and mutagenicity. Nevertheless, some Lepidoptera, including *Pachliopta aristolochiae*, feed exclusively on Aristolochiaceae and sequester AAs as a chemical defense. This is uncommon in nature and it is not yet fully understood how these insects avoid the lethal effects of AAs. To address this question, we investigate *Pac. aristolochiae*’s AA‐resistance mechanisms by employing metabolic analyses, multiomics analyses, in situ imaging and more. Our findings indicate that AAs may be detoxified through biotransformation and a robust antioxidant system, involving candidate genes such as 15‐oxo‐prostaglandin 13‐reductases (*PGRs*), cytochrome P450s, and catalases. Unexpectedly, DNA adducts, the covalent binding products from activated AA intermediates, are detected across most life stages of *Pac. aristolochiae*, revealing that *Pac. aristolochiae* can maintain genomic integrity despite a substantial burden (reaching over 1800 AA‐DNA adducts per 10^8^ nucleotides in adults, approximately 1 adduct per 55 000 nucleotides). Interestingly, however, no detectable DNA adducts are observed in wing discs, a representative organ undergoing metamorphosis, and AAs in testes are confined to somatic but not germ cells. Therefore, the strategies to protect against AA‐induced mutagenicity likely include restricted AA distribution in critical tissues and enhanced DNA repair. Using butterflies as an evolutionary model, we identify *PTGR1*, the human *PGR* homolog, as a potential target of AA resistance, which is associated with human acute kidney injury. Validation in human cells further demonstrates its role in reducing AA‐induced cytotoxicity and lipid peroxidation. Our study highlights insect AA tolerance as a means to discover human protective mechanisms, thereby suggesting new avenues for preventing AA‐related diseases.

## Graphical abstract

The study provides an extreme example of insect adaptation to highly toxic defenses of host plants, and investigates the complex strategies to resist carcinogenic aristolochic acids, including physical isolation, metabolic detoxification, and DNA repair. The study also reveals novel medical clues to human resistance to aristolochic acids inspired by insect evolution and adaptation.

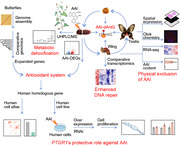

## 1 Introduction

Chemical defenses represent a ubiquitous form of protection in nature, encompassing a vast array of compounds with diverse biological roles. Plants, for instance, produce toxic chemicals to deter herbivores, yet some insects have evolved to not only tolerate these toxins but also weaponize them for their own defense [1, 2]. Humans, in turn, have harnessed these plant‐derived chemicals for medicinal purposes, often through empirical practices [3]. A striking example of this dynamic is found in the Aristolochiaceae family, which produces the phytotoxins known as aristolochic acids (AAs). Remarkably, some moth and butterfly species have evolved to feed exclusively on Aristolochiaceae and sequester AAs for their own defense against vertebrate predators [4, 5]. In humans, Aristolochiaceae have been used for millennia in traditional medicine [6], until their nephrotoxicity and carcinogenic effects were recognized in the late 20th century, prompting a reevaluation of their usage [7]. This exceptional adaptation in butterflies sparks a fascinating line of inquiry into how these insects circumvent the deleterious impact of AAs, which are notably toxic to mammals.

Due to growing concerns over the health risks of Aristolochiaceae, the mechanisms of their toxicity to mammals have been systematically studied. Aristolochic acid nephropathy (AAN) is a kidney disease caused by exposure to AAs, manifesting as acute kidney injury (AKI) or chronic progressive renal fibrosis [8], with severity dependent on exposure duration and dosage. Previous studies reveal that AAs induce renal tubular epithelial cell death through oxidative stress and apoptosis [9, 10]. In addition to their nephrotoxic effects, AAs are metabolically activated in vivo, producing DNA adducts and causing gene mutations that contribute to their carcinogenic effects [11]. Human exposure to AAs has been linked to urothelial cancers and liver premalignancy [12, 13]. Despite these well‐documented toxicities, humans continue to be exposed to AAs through traditional medicine and contaminated environmental media, including soil, water, and other crops [14, 15, 16, 17, 18]. As a result, AAN remains prevalent in Asia and the Balkans [19], and the mutational signature of AAs is increasingly being reported in human tumor genomic sequencing data [12, 13, 20]. While current strategies for managing these health risks primarily utilize DNA adducts as biomarkers for early diagnosis [11], the treatment is still mainly focused on alleviating symptoms. Previous studies have shown that antioxidants may mitigate AA‐induced injury in experimental systems [21, 22, 23], but no therapies currently target the underlying toxicity. Thus, the absence of mechanism‐based treatments highlights an urgent need for innovative approaches. Therefore, studying insects that have evolved to resist AAs may provide key insights and offer novel solutions for mitigating their harmful effects in humans.

During the long‐term coevolution with host plants, multiple herbivorous insects (e.g., most moth and butterfly larvae) have evolved a variety of mechanisms to cope with toxic phytochemicals, including terpenes and nitrogen‐containing compounds [24]. These strategies encompass physiological adaptations, such as the mutation of Na^+^/K^+^‐ATPase in the monarch butterfly to counteract cardiac glycosides [25], and the enzymatic transformation of phytochemicals into less toxic compounds, such as the conversion of tertiary pyrrolizidine alkaloids to their *N*‐oxides [26]. However, compared to these well‐characterized detoxification mechanisms, the resistance to AAs in butterflies remains poorly understood.

In mammals, the metabolism of AAs is characterized by a dual outcome: detoxification versus activation. Specifically, aristolochic acid I (AAI) can undergo oxidative *O*‐demethylation (catalyzed by *CYP1A1/2*) to the less toxic AAIa [27, 28], which is subsequently conjugated by the phase II enzymes and eliminated renally [29, 30], representing a critical detoxification route that mitigates nephrotoxicity and genotoxicity. In an alternative pathway, AAI can also undergo nitroreduction, primarily mediated by quinone oxidoreductase (*NQO1*) [31], yielding *N*‐hydroxyaristolactam I (*N*‐OH‐ALI). While *N*‐OH‐ALI may be further metabolized to the inactive forms such as aristolactam I (ALI) or 7‐hydroxyaristolactam I (7‐OH‐ALI), it also acts as a precursor for bioactivation [32]. The resulting reactive intermediates covalently bind purine nucleotides in DNA, yielding persistent adducts (e.g., dA‐AAI/dG‐AAI) that drive mutations and promote carcinogenesis. In the Aristolochiaceae‐feeding butterfly *Battus polydamas*, the presence of AAIa [33] and AA *O*‐glucosides [34] provides evidence that AAs may undergo biotransformation, although the exact metabolic processes are not elucidated. On the other hand, whether mammalian‐like bioactivation pathways exist remains to be determined, as key metabolites like ALI have not been detected in some studies [34], or when detected, has been attributed to host plant origins [35].

Consequently, it remains uncertain whether biotransformation during AA detoxification also results in DNA adducts, and if so, how do these insects protect against DNA lesions to ensure genomic and developmental stability? In addition to combating potential DNA damage, insects must counteract AA‐induced oxidative stress and mitochondrial dysfunction—mechanisms known to trigger renal injury in mammals [9, 10]. This raises another critical question: how do these insects mitigate oxidative toxicity? Given these challenges, exploring the rare survival strategies of these insects may uncover novel mechanisms of resistance of AAs, inspiring innovative medical therapies, such as targeted treatments for AA‐induced nephrotoxicity and carcinogenicity.

To address these questions, we systematically investigated the anti‐aristolochic acid mechanisms in *Pachliopta aristolochiae* (Papilionidae: Papilioninae: Troidini), whose larvae feed exclusively on *Aristolochia* species. First, we treated butterfly larvae with exogenous AAI and characterized its biotransformation and resulting metabolites. Next, we examined DNA adduct formation across all life stages and in key organs. To elucidate the genetic underpinnings of AA resistance, we combined comparative genomic analyses of *Pac. aristolochiae* and other non‐toxic butterfly species with transcriptomic analyses, identifying candidate genes for conferring AA resistance, i.e., specifically expanded gene families, highly expressed genes, and genes that were differentially expressed in response to AAI. Furthermore, we investigated protective strategies in development‐ and reproduction‐related organs (e.g., wing discs and testes) through AAI concentration measurement, in situ visualization, and multi‐omics analyses. Finally, guided by the AA response genes in butterflies and their potential antioxidant roles, we focused on the functions of their human homologs, with particular attention to *PTGR1*. We conducted a meta‐analysis of human single‐cell multi‐omics data to verify the association of *PTGR1* expression with AKI and verified its protective potential against AA‐induced toxicity in human cells. In summary, this study leveraged the AA tolerance mechanisms of *Pac. aristolochiae* to uncover protective pathways, thereby providing novel insights into toxin resistance and potential strategies for mitigating AA‐related health risks in humans.

## 2 Results

### 2.1 Metabolic Detoxification of AAI and DNA Adducts in *Pac. aristolochiae*

**FIGURE 1:**
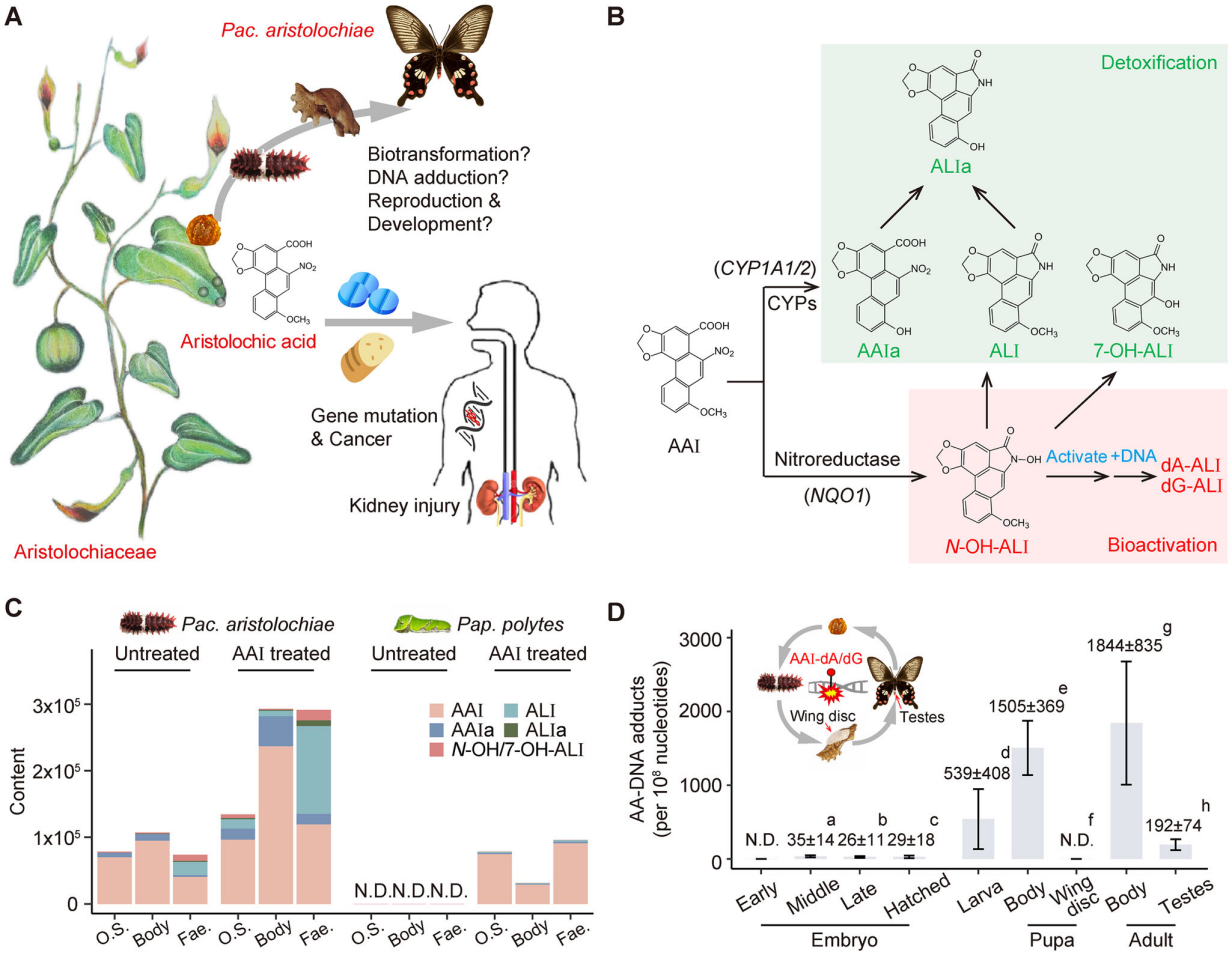
Biotransformation and DNA adduction of AAI by *Pac. aristolochiae*. (A) *Aristolochia* spp. are carcinogenic and nephrotoxic in humans but are consumed by *Pac. aristolochiae* larvae. (B) The in vivo pathway of AAI metabolism according to findings from published studies in humans and rodents is summarized. AAI is oxidized to AAIa by cytochrome P450 genes (CYPs) and reduced to *N‐*hydroxyaristolactam I (*N‐*OH‐ALI) by nitroreductase. *N‐*OH‐ALI is converted to aristolactam I (ALI) by further reduction or to 7‐hydroxyaristolactam I (7‐OH‐ALI) by Bamberger rearrangement. Both the oxidative demethylation of ALI and the nitroreduction of AAIa lead to the development of ALIa. *N‐*OH‐ALI can also be dehydroxylated into highly electrophilic cyclic *N‐*acylnitrenium ions to form DNA adducts. (C) Estimated levels of AAI, AAIa, ALI, ALIa, and *N‐*OH/7‐OH‐ALI in the larval body, osmotic fluid (O.S.) and feces (Fae.) in *Pac. aristolochiae* and *Pap. polytes* with and without AAI treatment. (D) Quantitative analysis of AA‐DNA adduct formation was conducted throughout all life stages of *Pac. aristolochiae*, also including pupal wing discs, and adult testes. Middle‐stage eggs (3–5 days after oviposition) were pooled for DNA isolation and adduct detection, with a total of 27, 37, and 14 eggs pooled in different tests. The plot shows the mean ± SD of 3 measurements (a). Larvae were dissected from the eggs at the latest embryonic stage just before hatching, with 26 larvae pooled for detection. The mean ± SD of 2 repeated measurements is shown (b). Egg shells were washed to remove AAs, and 26 and 13 newly hatched first instar larvae were pooled for detection. The plot shows the mean ± SD of 2 measurements (c). The mean ± SD from 7 untreated L5 larvae is shown (d). The mean ± SD from 8 untreated pupae is shown (e). Wing discs were isolated from 7 pupae in independent tests, and no DNA adducts were detected (f). Adult butterflies without legs and wings were used for detection. The mean ± SD from 7 individuals is shown (g). Testes from 4 and 8 adults were pooled for detection. The mean ± SD of 2 repeated measurements is shown (h).

Our study aimed to understand how *Pac. aristolochiae* tolerates AAs, toxins that are harmful to humans but are abundant in the host plants of this butterfly (Figure 1A). To investigate whether AAs are detoxified through biotransformation, we first examined the disposition and metabolism of AAs in *Pac. aristolochiae* by conducting comparative experiments with a sympatric, Rutaceae‐feeding butterfly species in the same subfamily, *Papilio polytes*. *Pap. polytes* females are polymorphic, Batesian mimics of a few well‐defended model species, including *Pac. aristolochiae* [36, 37].

We conducted experiments on fifth‐instar larvae from both species under two conditions: a control group fed with normal host plants, and an experimental group treated with exogenous AAI, by mixing a solution of AAI into their diet at a dosage of > 100 mg/kg body weight (Figures S1 and S2). The compound used, AAI, is the most abundant and well‐studied AA analog in *Aristolochia debilis*, the host plant of *Pac. aristolochiae* at our field site. We subsequently traced and compared the biotransformation of AAI in *Pac. aristolochiae* and *Pap. polytes* by profiling the major metabolites. Their relative amounts were estimated based on the peak intensities obtained from high‐resolution mass spectrometry (Figure 1C; Figure S3, and Table S1).

*Pap. polytes* larvae consuming AAI‐treated leaves showed normal behavior and primarily excreted the unmetabolized compound. The absence of detectable active intermediates further indicates that neither metabolic activation nor detoxification of AAI was induced (Figure 1C). In contrast, additional exposure to AAI resulted in a notable accumulation of AAI in *Pac. aristolochiae* larvae, particularly in the body tissues. Concurrently, the elevated levels of the metabolites AAIa and ALI in fecal samples suggest that AAI was metabolized and subsequently excreted, providing evidence that both oxidative detoxification and reductive activation pathways of AAs were involved (Figure 1B,C).

To further determine whether the potential activation of AAs leads to DNA adduct formation, we quantified adduct levels throughout the life stages of *Pac. aristolochiae* (Figures S4 and S5). As shown in Figure 1D, the levels of DNA adducts in fertilized eggs were extremely low, with fewer than 50 adducts per 10^8^ nucleotides in the middle and late stages, whereas the levels increased to > 500 adducts per 10^8^ nucleotides at the larval stage and continuously increased in pupae and adults, reaching approximately 1800 adducts per 10^8^ nucleotides in adults. Given the high levels of adducts detected in the entire bodies of pupae and adults, we became curious about the AAI levels in key developmental and reproductive organs that may be crucial to the completion of the butterfly's life cycle. Therefore, we further determined the degree of DNA adduct formation in the pupal wings and testes of male adults. Surprisingly, AAI‐DNA adducts were not detected at all in dissected pupal wings and were detectable in adult testes, although at levels approximately ten times lower than those in the adult body (Figure 1D).

### 2.2 Gene Expansion and Differential Expression Associated with Metabolism and Oxidative Stress

**FIGURE 2:**
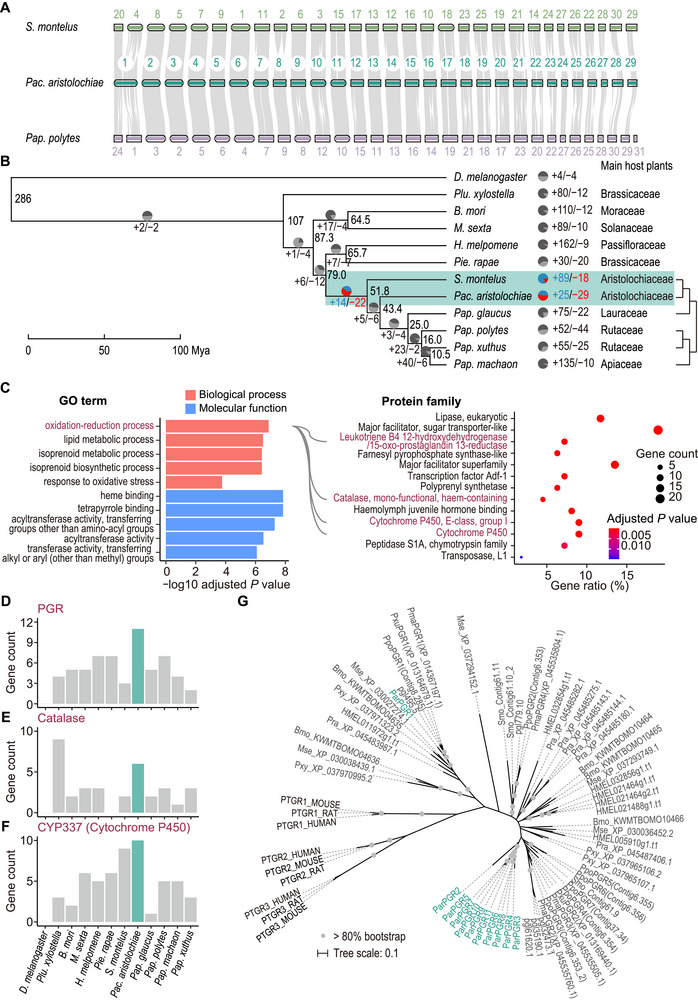
Comparative genomic analysis of Papilionidae butterflies. (A) Syntenic relationships among *Pac. aristolochiae*, *S. montelus*, and *Pap. polytes* chromosomes. Each line represents a syntenic block between two species. (B) The phylogeny and primary host plant families of focal taxa. The time‐calibrated phylogenetic tree was reconstructed from protein alignments of 2954 single‐copy genes of 11 Lepidoptera species with *D. melanogaster* as the outgroup. The numbers at the nodes represent the divergence time (million years ago, Mya). The pie chart at each branch represents the number of significantly expanded and contracted gene families (branch‐specific *p* < 0.01). The Papilionidae that feed on Aristolochiaceae are highlighted in green. (C) Functional and structural enrichment of expanded gene families in *Pac. aristolochiae* genome. The top five most significant GO terms and the significantly enriched protein families are displayed (hypergeometric test with FDR correction, adjusted *p* < 0.05). The size of the dots represents the number of genes in the protein family, and the color represents the significance. The lines connecting protein families overlap with the focal function entry. The bar plots show the gene numbers of candidate protein families, namely, *LTB4 12‐HD/PGR* (D), *catalase* (E) and *CYP337* (F), in each species. (G) Phylogenetic tree of the PGR gene family. The *ParPGR1* group has one copy per species. *ParPGR2–11* clustered together and diverged from *PGR* genes of other Lepidoptera. Three mammalian *PTGR* genes were also included in the analysis.

To further elucidate the genetic mechanisms underlying AA resistance in *Pac. aristolochiae*, we constructed a high‐quality reference genome of *Pac. aristolochiae* at the chromosome level, with a size of approximately 315.3 Mb and a BUSCO completeness of 98.6% (Figure 2A; Figure S6). We annotated a total of 12 438 protein‐coding genes that contained 93.2% of the insect‐conserved genes and inferred the phylogenetic relationships of 11 Lepidoptera species and *Drosophila melanogaster* using 2954 single‐copy genes, revealing a monophyletic group including *Pac. aristolochiae* and the genus *Papilio*. Another Aristolochiaceae‐feeding swallowtail butterfly, *Sericinus montelus*, was the earliest diverging lineage included in our sample and diverged from the other species approximately 52 million years ago (Figure 2B; Figure S7), which was broadly consistent with previous results [38].

We first conducted comparative analyses to characterize rapidly evolving gene families in *Pac. aristolochiae* and identified 25 significantly expanded gene families (branch‐specific *p* < 0.01) involved in the oxidation‐reduction process and lipid metabolic processes (hypergeometric test adjusted *p* < 0.05, Figure 2B,C). More specifically, the genes involved in the oxidation‐reduction process belong to the *15‐oxo‐prostaglandin 13‐reductases* (*PGR*), *catalase*, and *cytochrome P450* (*CYP*) gene families (Figure 2C). The *CYP* genes, in particular, encode phase I metabolic enzymes that mediate the initial detoxification step through biotransformation of toxic compounds. Specifically, these *CYP*s belong to the insect *CYP3* clan, which are homologs of *CYP337* in silkworms (*Bombyx mori*) and form two dispersed gene clusters, likely owing to increases in gene copy number and chromosomal translocations (Figure 2F; Figure S8D). In contrast, other expanded gene families may not be directly involved in AA metabolism. The *PGR* genes encode members of the medium‐chain dehydrogenases/reductases and alkenal/one oxidoreductases, which participate in eicosanoid catabolism and lipid peroxidation [39], suggesting a potential role in oxidative stress defense in *Pac. aristolochiae*. Notably, we identified 11 *PGR* genes in *Pac. aristolochiae*, which is far more than in other lepidopteran species, while *D. melanogaster* completely lacks *PGR* genes (Figure 2D). Among these *ParPGR* genes, a gene located on chromosome 14 (*ParPGR1*) was highly conserved in Lepidoptera species. The remaining *PGR*s formed two gene clusters on chromosome 10 (*ParPGR2–11*) and originated after the divergence between *Pac. aristolochiae* and other closely related taxa (Figure 2G; Figure S8B). Importantly, these *Pac. aristolochiae*‐specific *PGR*s showed significantly increased Ka/Ks ratios compared to the conserved *ParPGR1* (Wilcoxon rank sum test, *p* = 4 × 10^−6^, Figure S8A), indicating possible functional differentiation resulting from different selective pressures. Similarly, the *catalase* gene family, which breaks down hydrogen peroxide in organisms and plays a crucial role in antioxidant defense, was significantly expanded in *Pac. aristolochiae* such that this species contains more *catalase* genes than any other Papilionidae species in our analysis (branch‐specific *p* < 0.01, Figure 2E). However, unlike the *PGR*s, the *catalase* genes did not form multicopy clusters and may be differentially regulated (Figure S8C,E).

Since Aristolochiaceae is recognized as the ancestral host of Papilionidae butterflies [40], we further investigated gene family evolution in the basal lineage of Papilionidae to explore the possible genomic changes associated with the feeding habit, which yielded 14 significantly expanded gene families (branch‐specific *p* < 0.01) that were also enriched in the oxidation‐reduction process, including mainly *CYP* and *short‐chain dehydrogenase*/*reductase* (*SDR*) genes (hypergeometric test adjusted *p* < 0.05, Figure S9). More specifically, these expanded *CYP*s belong to the gene families *CYP6AU1* and *CYP324A1* of *CYP3*, and both *CYP* families also experienced species‐specific gene duplications in *Pac. aristolochiae* (Figure S10). In contrast, the gene copy numbers of *CYP2*, *CYP4*, and mitochondrial *CYP* clans remained relatively conserved (Figure S11). In addition to the *CYP* genes, we also found that the phase II metabolic enzyme *glutathione S‐transferase* (*GST*), specifically the *GSTD* subfamily, also expanded in the Papilionidae ancestors (branch‐specific *p* < 0.01, Figures S9 and S12). To summarize, our results showed that gene families represented by *CYP* and *PGR* evolved rapidly during the origin and differentiation of Papilionidae. Since AA‐induced toxicity in mammals is associated with oxidative stress [9, 10], we hypothesized that these specifically expanded gene families may represent an adaptive mechanism for antioxidant defense and the detoxification of secondary metabolic compounds.

**FIGURE 3:**
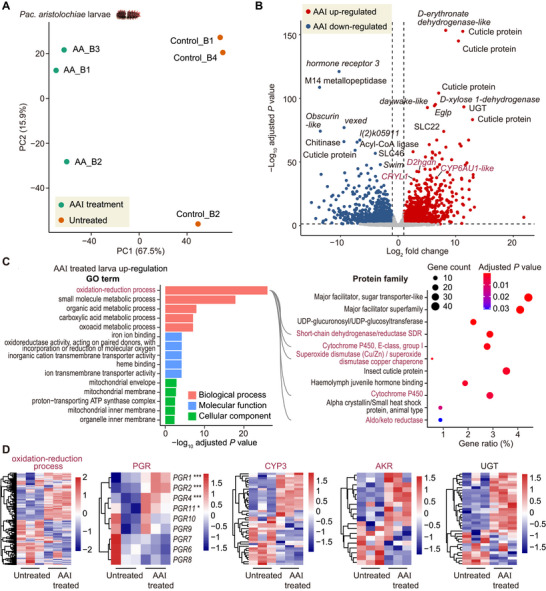
Transcriptome changes induced by AAI in *Pac. aristolochiae*. (A) Principal component analysis of AAI‐treated and untreated samples. Each group included three biological replicates. (B) Volcano plot showing the genes with significant upregulation (red) and downregulation (blue) after AAI treatment (Wald test with FDR correction, adjusted *p* < 0.05). (C) Functional and protein family enrichment of upregulated genes in AAI‐treated *Pac. aristolochiae* larvae. The top five most significant GO terms are displayed (hypergeometric test with FDR correction, adjusted *p* < 0.05). The lines connecting protein families overlap with the focal function entry. (D) Normalized and scaled expression levels of the focal gene families in AAI‐treated and untreated *Pac. aristolochiae* larvae. * indicates differential expression with an adjusted *p* of < 0.05, and *** indicates an adjusted *p* < 0.001 (Wald test with FDR correction).

To explore whether rapidly evolving genes are responsible for handling AAs to confer toxicity tolerance and, if so, how they integrate and function with other genes, we further fed the larvae with normal diets or diets containing additional AAI and compared the gene expression patterns of the two groups (Figure 3). Principal component analysis (PCA) revealed that the AAI‐treated samples were separated from the control samples on the basis of their gene expression (Figure 3A), indicating a large number of differentially expressed genes (DEGs) between the groups (Wald test adjusted *p* < 0.05, Figure 3B). Notably, the 1447 genes whose expression was upregulated in response to AAI treatment were functionally enriched in oxidation‐reduction process and belonged mainly to the *CYP*, *SDR*, and the *aldo/keto reductase* (*AKR*) gene families (hypergeometric test adjusted *p* < 0.05, Figure 3C,D). Echoing the results above, we also found multiple upregulated *PGR*s associated with the oxidation‐reduction process among the gene families enriched by AAI induction (*p* = 0.02, adjusted *p* = 0.13; Figure 3D), further suggesting that their increased copy number may be a specific response to AA toxicity. In addition, most of these differentially expressed *CYP*s (20 of 26 genes) were from the rapidly evolving *CYP3* clan. Given that AAI also undergoes nitroreduction to produce *N*‐OH‐ALI, a core metabolite that converts AAI into ALI or 7‐OH‐ALI for detoxification (Figure 1B), we propose that the reductases we identified (*SDRs* and *AKRs*) may also contribute to the first‐phase biotransformation of AAs. Moreover, we noted that the *UDP‐glucosyltransferase* (*UGT*) genes were also significantly enriched in AA‐response genes (hypergeometric test adjusted *p* < 0.05, Figure 3C,D), which encode primary phase II enzymes that catalyze the conjugation of glucuronic acid to xenobiotics with polar groups, e.g., human *UDP1*, which was reported to catalyze AA analogs into less nephrotoxic glucuronides [41]. In addition to oxidoreductases, the *major facilitator superfamily* (*MFS*), identified as AA‐response transporters and represented by the *solute carrier family 22* genes (*SLC22*, encoding organic ion transporters), was the most significantly enriched gene family among the upregulated genes (hypergeometric test adjusted *p* < 0.05, Figure 3C). Taken together, our findings suggest that these enzymes and transporters whose expression was upregulated by AAI treatment are likely involved in combating AA‐induced oxidative damage and in the metabolism and passage of AAs out of the body to confer resistance.

### 2.3 Physical Isolation, Low Metabolic Activation, and Enhanced DNA Repair as Multi‐Layered Anti‐AA Strategies

**FIGURE 4:**
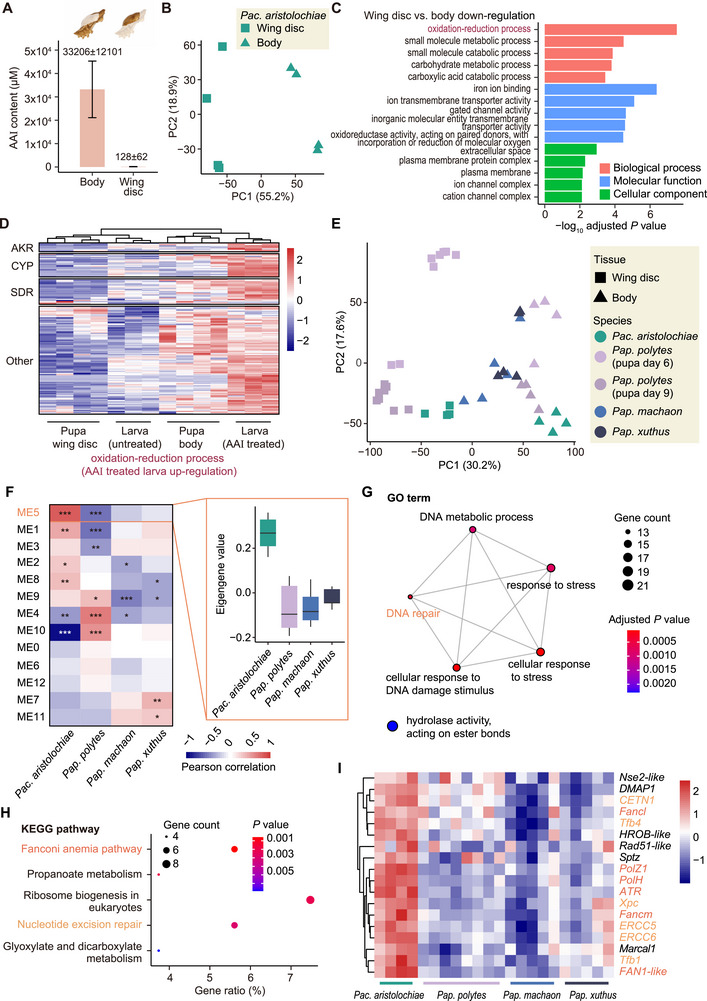
Comparative transcriptomic analysis of Papilionidae butterflies. (A) AAI concentration in the *Pac. aristolochiae* pupa wing discs and body. The parts used for testing are shown in color. The means ± SDs of *n* = 4 individuals are shown. (B) PCA of *Pac. aristolochiae* pupa wing disc and body transcriptome data. (C) Functional enrichment of downregulated genes in *Pac. aristolochiae* pupa wing discs. The top five most significant GO terms are displayed (hypergeometric test with FDR correction, adjusted *p* < 0.05). (D) Normalized and scaled expression levels of exogenous aristolochic acid response genes related to the oxidation‐reduction process in *Pac. aristolochiae* larva and pupa. (E) Principal component analysis results for all samples. The color of the point represents the species, and the shape represents the tissue. *Pap. polytes* samples from two stages are included in the analysis. The log‐transformed FPKM values are used as the input. (F) Relationships between modules in gene co‐expression networks and four species of Papilionidae butterflies. The heatmap shows the correlations between module eigengenes (MEs) and species. The gene expression profile of module 5 had the strongest positive correlation with *Pac. aristolochiae*. Student asymptotic *p* values for given correlations were calculated, where * indicates *p* < 0.05, ** indicates *p* < 0.01, and *** indicates *p* < 0.001. The box plot shows the ME value of module 5 for each species. (G) Functional enrichment of module 5 hub genes (correlations between gene expression and module eigengenes > 0.8). All significant terms are shown (hypergeometric test with FDR correction, adjusted *p* < 0.05). The size of the dots represents the number of genes, and the color represents the significance. (H) KEGG enrichment of module 5 hub genes. KEGG pathways with a *p* < 0.01 (hypergeometric test) are displayed. (I) Normalized and scaled expression levels of module 5 genes related to DNA repair, the Fanconi anemia pathway, and the nucleotide excision repair pathway. The samples of the pupal bodies of the four species are shown in the heatmap.

Given the detection of metabolic activation of AAs and widespread DNA adduct formation in *Pac. aristolochiae*, we sought to investigate how these butterflies protect their critical reproductive and developmental organs from DNA damage. We focused on pupal wing discs as representative organs during development, as wings are critical for butterfly survival, and exposure to mutagens during development may affect adult wing function. Surprisingly, while high levels of DNA adducts were detected throughout the pupae, no detectable AAI‐DNA adducts were observed in the wing discs (Figure 1D). To elucidate whether AAs are separated between the body and wing discs, we determined the exact AAI concentrations in both tissues and found that AAI was present in the wing discs at an extremely low level—approximately 260 times lower than that of the body (Figure 4A). This suggests a mechanism that effectively prevents AAs from entering the wing discs, which may be achieved through a physical barrier such as the glassy peripodial membrane separating the wing disc from the body. However, the presence of trace amounts of AAI in the wing discs, despite the absence of DNA adducts, implies that other protective mechanisms are still needed. To explore this further, we compared gene expression patterns between wing discs and body tissues, revealing a tissue‐specific expression pattern: 1805 genes were significantly upregulated in the body, compared to only 808 in the wing discs (Wald test adjusted *p* < 0.05, Figure 4B; Figure S13A). Notably, genes downregulated in wing discs were enriched in oxidation‐reduction process, including 28 *SDR* genes and 5 *AKR* genes (Figure 4C; Figure S13B,C), which were upregulated as responsive genes in AAI‐treated larvae (Figure 3C). Similarly, the phase II metabolic enzyme *UGT* genes also presented relatively low expression in wing discs (Figure S13B,C). This low expression of metabolic enzymes in wing discs likely prevents AA activation, explaining the absence of detectable DNA adducts despite trace levels of AAs (Figure 4D). We also observed lower expression of *SLC22* genes, potential AA transporters, in wing discs compared to body tissues (Figure S13B). Specifically, 34 of the 79 *SLC22* genes were significantly downregulated versus only five that were upregulated (Wald test adjusted *p* < 0.05, Figure S13C), suggesting that, in addition to physical isolation, reduced expression of the transporters may further prevent AA uptake into the wing discs.

Overall, the combination of physical separation and reduced bioactivation of AAs may act as a ‘belt and braces’ approach to protect wing disc DNA from damage. However, given the high levels of AAs and DNA adducts detected in body tissues, we investigated whether *Pac. aristolochiae* possesses unique gene expression features to mitigate mutation risks and maintain physiological functions. To address this, we compared the expression profile of *Pac. aristolochiae* with that of *Pap. polytes*, which does not feed on Aristolochiaceae (Table S2). The PCA results revealed that the samples were clustered primarily by tissue type rather than species, suggesting that both tissue and species traits should be considered (Figure S14A,B). We then constructed gene co‐expression networks, which classified genes into 10 gene modules on the basis of expression pattern similarity (Figure S14C,D). We further calculated the correlations between the module expression profiles (module eigengenes) and the biological traits to identify highly expressed genes in target tissues or species. Module 2 was the only module significantly correlated with body tissue across species and developmental stages (Student asymptotic *p* < 0.05, Figure S14E). In module 2, a total of 1530 genes were enriched for transmembrane transport and oxidation‐reduction processes (Figure S15A), suggesting a conserved pattern of body oxidation‐reduction that may be relevant to general herbivory responses, regardless of whether Aristolochiaceae is consumed. In addition, module 1 comprised 3056 genes that were highly expressed in *Pac. aristolochiae*, and the hub genes in this module were related mainly to the DNA repair process and the nucleotide excision repair pathway (Figure S15B–D), suggesting that DNA repair is particularly important for *Pac. aristolochiae*.

To avoid bias in the detection of genes with low expression in *Pap. polytes*, we also collected and analyzed transcriptome data from the pupal stage from two additional *Papilio* species as the control group (Table S2). Consistent with previous results, the PCA results reflected a tissue‐specific clustering pattern, and 12 gene modules were identified on the basis of the constructed gene co‐expression networks (Figure 4E,F; Figure S16). Among them, four modules showed significant positive correlations with *Pac. aristolochiae* (Student asymptotic *p* < 0.05); the highest correlation was for module 5, with a coefficient of 0.83 (Figure 4F). Coincidentally, module 5 also showed the highest negative correlation with *Pap. polytes* (Figure 4F), and it was also the only module significantly positively correlated with both the body and wing disc tissues of *Pac. aristolochiae* (Student asymptotic *p* < 0.05, Figure S16D). Consistent with the results of the correlation analysis, the eigengenes of module 5 were relatively higher in *Pac. aristolochiae* than other species (Figure 4F), suggesting that the genes in module 5 were highly expressed in Aristolochiaceae‐feeding species. Notably, among the 495 genes in module 5, there were 284 hub genes significantly enriched in DNA damage repair, particularly in the Fanconi anemia (FA) and nucleotide excision repair (NER) pathways (hypergeometric test *p* < 0.05, Figure 4G,H). Therefore, the results of our network analysis further confirmed that a set of genes related to DNA repair is specifically and highly expressed in *Pac. aristolochiae* (Figure 4I; Figure S17), which may play a key role in counteracting AA‐induced DNA damage and ensuring physiological functions.

**FIGURE 5:**
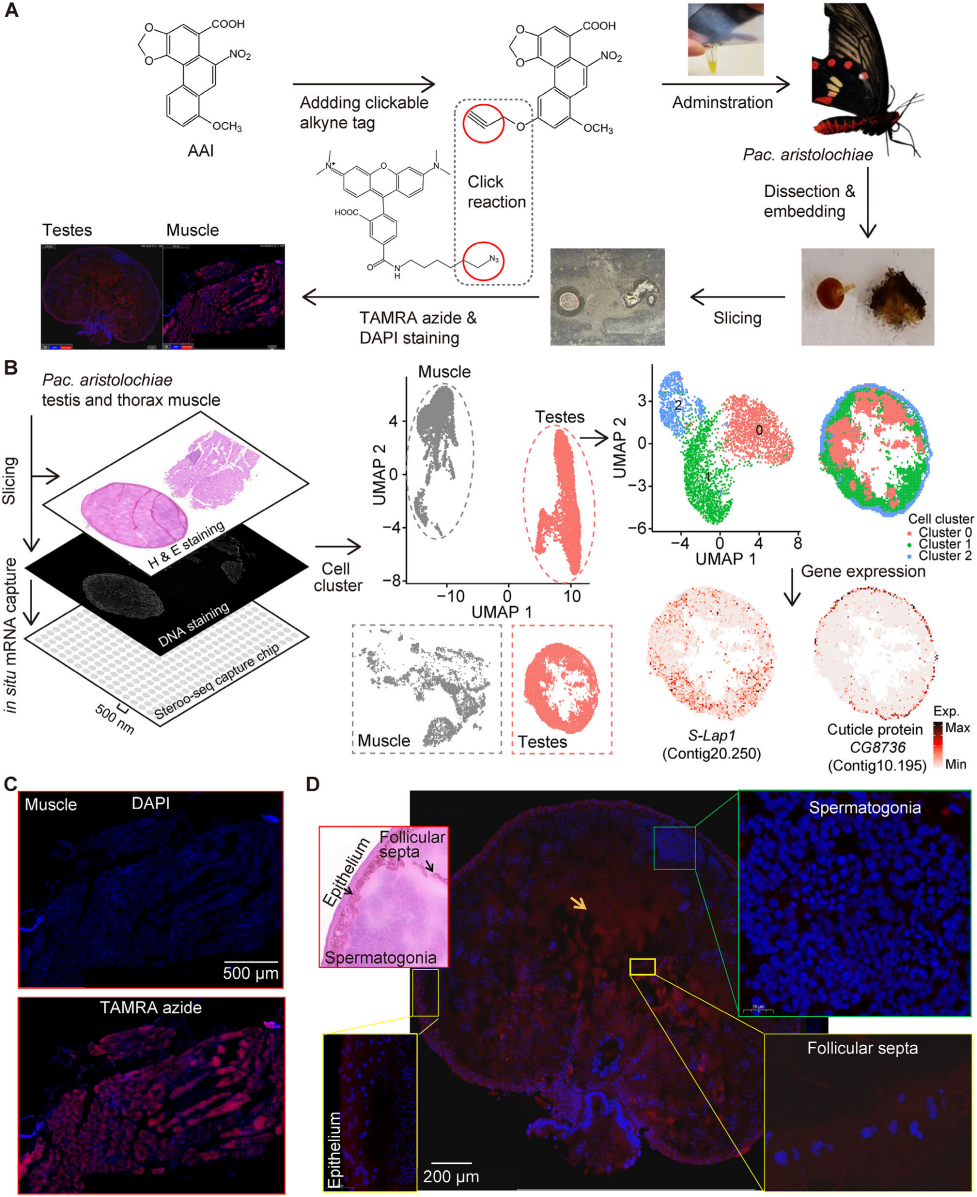
In situ AAI detection and spatial transcriptomic analysis in the adult *Pac. aristolochiae* testis and thorax. (A) Schematic approach for click chemistry‐based in situ detection of the monoalkyne AAI. (B) Stereo‐seq spatial transcriptome profiling for the identification and annotation of distinct cell types. The left schematic diagram shows the H&E and DNA staining of the tissue sections. The middle plot shows the clustering results based on the UMAP algorithm and the spatial positions of the testis and thorax muscle cells. The cell subsets of the testis and the spatial expression patterns of major marker genes in the testis, including the *S‐Lap* gene *Contig20.250* and the cuticle protein‐encoding gene *Contig10.195*, are plotted. Darker colors represent higher expression levels. (C) Distribution of the monoalkyne AAI in thorax tissue. The blue fluorescence is from DAPI for the DNA stain (left), and the red fluorescence is from TAMRA azide from the monoalkyne AAI on the basis of the click chemistry reaction (right). (D) Distribution of the monoalkyne AAI in testis tissue.

In addition to protecting against AAs during individual development, safeguarding the reproductive system from AAs is crucial. When we examined DNA adduct formation in adult *Pac. aristolochiae*, we were surprised that AAI‐DNA adducts were also detectable in the testes, albeit at a low level (Figure 1D), raising the question of how these insects protect their germline from mutagenic AAs. Since the testis comprises both somatic cells and germ cells, we then investigated whether AAs could enter germ cells. To visualize the spatial distribution of AAI, we designed and synthesized an alkyne‐tagged AAI (Figure S18) and administered it to an adult *Pac. aristolochiae* (Figure 5A; Figure S19). Using a copper‐catalyzed click reaction, we visualized the spatial distribution of monoalkyne AAI via red‐fluorescent TAMRA azide staining. Moreover, we performed single‐cell resolved spatial transcriptomic analysis to differentiate germ cells and somatic cells on the basis of their spatial features and identify genes expressed in specific tissues (Figure 5B). Cells in testis and thoracic tissues were divided into two clusters by tissue type (Figure 5B; Figure S20), and the testis cells could be further divided into three subsets (Figure S21). Among them, cell subset 2, which had the smallest number of cells, comprised cells from the edge of the testis that expressed high levels of a cuticle protein‐encoding gene and was considered to represent epithelial cells (Figure 5B). In contrast, cell subsets 0 and 1 corresponded to potential germline cells. Cell subset 1 expressed the sperm leucyl aminopeptidase gene (*S‐Lap1*) and was recognized as spermatids. Cell subset 0 was characterized by high expression of *Rim*, a marker gene specific to spermatocytes in *D. melanogaster* (Figure 5B; Figure S21). At 18 h after administration, alkyne‐tagged AAI was evenly distributed in thorax tissue cells, and its intracellular uptake was revealed by the co‐localization of DAPI (blue) and the TAMRA azide‐labeled monoalkyne AAI (red) (Figure 5C). In the testes the monoalkyne AAI entered the interior of the testis and dispersed in the interstitial fluid (arrow in Figure 5D), but only in the coating epithelium and follicular septa, shown as the deeply H&E stained area (top‐left panel of Figure 5D), and co‐staining with DAPI and TAMRA revealed the intracellular distribution of the monoalkyne AAI in those somatic cells (bottom‐left and bottom‐right panels of Figure 5D). In contrast, the lack of TAMRA staining in the spermatogonial area (top‐right panel of Figure 5D) indicates that monoalkyne AAI did not accumulate in germ cells, suggesting the presence of a selective barrier that restricts AAI uptake or ensures its efficient export. To explore this further, we identified a transporter gene highly expressed in germline cells, *Contig8.251*, which is a homolog of *CG8654* in *D. melanogaster* and belongs to the *SLC22* gene family. Meanwhile, it exhibits high sequence and structure similarity to the known human AA transporters, the organic cation transporters (OATs) [42] (Figures S21C and S22), indicating that it may serve as a barrier against the accumulation of xenobiotics into the reproductive system.

### 2.4 The Human Homolog of the Butterfly PGR Gene Protects Against AAI‐Induced Toxicity in Human Cells

**FIGURE 6:**
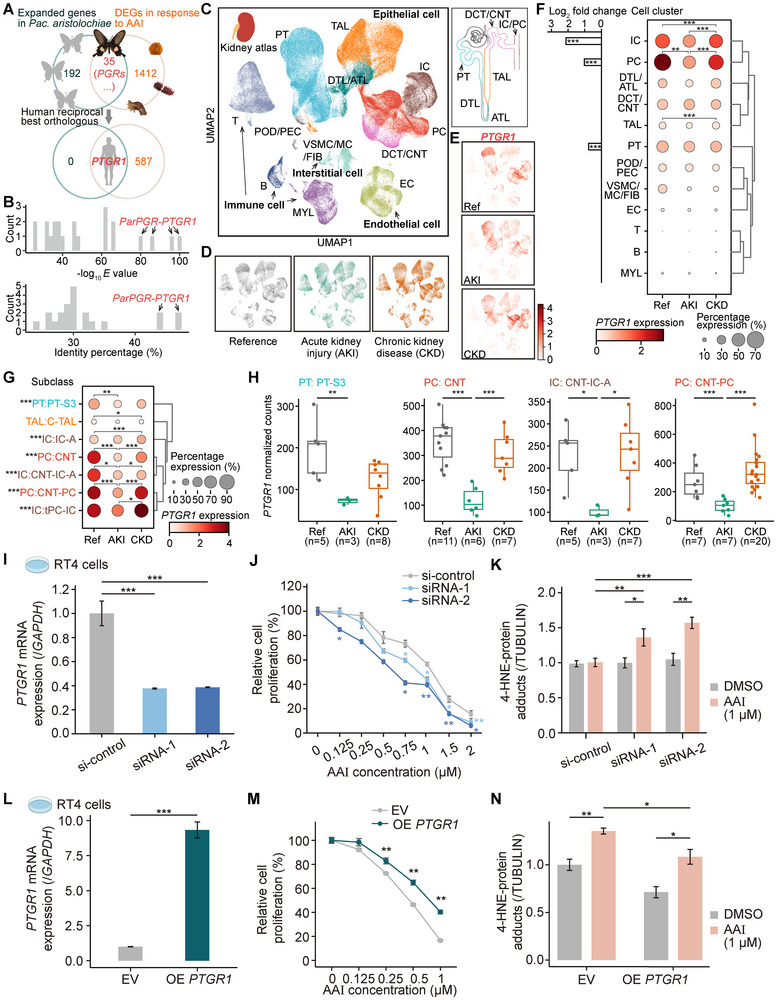
Identification of *PGRs* and the function of human *PTGR1* in AAI‐induced toxicity. (A) Venn diagrams illustrating the number of overlapping genes between expanded and AAI‐induced gene sets in *Pac. aristolochiae*, as well as the number of corresponding homologous genes identified in the human genome. (B) Histogram plots showing the distribution of *E* values and the amino acid identity between the previously described intersecting gene sets and their best human hits. (C) The UMAP embedding of human kidney single‐cell atlas. The color represents the cell cluster. The schematic diagram of the nephron shows the structure and position of the renal tubule epithelial cells. PT, proximal tubule; DTL, descending thin limb; ATL, ascending thin limb; TAL, thick ascending limb; DCT, distal convoluted tubule; CNT, connecting tubule; PC, principal cell; IC, intercalated cell; POD, podocyte; PEC, parietal epithelial cell; VSMC, vascular smooth muscle cell; MC, mesangial cell; FIB, fibroblast; EC, endothelial cell; MYL, myeloid cell; T, T cell; B, B cell. (D) The distribution of cells derived from healthy reference (Ref) and acute kidney injury (AKI) and chronic kidney disease (CKD) patients. (E) The expression patterns of *PTGR1* in the UMAP reduction. Darker colors represent higher expression levels. (F) The dot plot shows the percentage and average expression level of *PTGR1* across all cell clusters in control and disease conditions. Differential expression analysis between disease groups was performed based on the pseudobulk method (Wald test with FDR correction). The bar plot on the left shows the expression fold change between each cell cluster and the rest of the cells. Only significant and positive expression differences are plotted (Wilcoxon rank sum test with FDR correction). (G) The percentage and average expression level of *PTGR1* in cell subclasses. Only subclasses with significant expression differences between groups are shown (Wald test with FDR correction). Comparison results across cell subclasses are labeled on the left (Wilcoxon rank sum test with FDR correction). (H) Normalized counts of *PTGR1* in reference and disease samples in the specific cell subclasses (Wald test with FDR correction). * indicates adjusted *p* < 0.05, ** indicates adjusted *p* < 0.01 and *** indicates adjusted *p* < 0.001 in F, G, H. (I) RNA interference of *PTGR1* in RT4 cells. Two siRNAs are used, and the plot shows the relative expression levels of *PTGR1*, with *GAPDH* as the internal control gene. (J) Relative cell proliferation rates of *PTGR1*‐knockdown cells treated with different concentrations of AAI. (K) The production of 4‐HNE‐protein adducts induced by 1 µm AAI in *PTGR1*‐knockdown cells was detected via western blotting after a 24 h treatment period. (L) The expression levels of *PTGR1* mRNA after transfection with *PTGR1* plasmid (OE *PTGR1*) or empty control vector (EV), with *GAPDH* as the internal control gene. (M) Relative cell proliferation rates of *PTGR1*‐overexpression cells treated with different concentrations of AAI. (N) The production of 4‐HNE‐protein adducts induced by 1 µm AAI in *PTGR1*‐overexpression cells. * indicates *p* < 0.05, ** indicates *p* < 0.01, and *** indicates *p* < 0.001 in I, K, L, N (*T*‐Test) and J, M (Wilcoxon rank sum test). The data are expressed as the mean ± SEM.

Inspired by our butterfly studies in view of the risks that AAs pose to human health, we explored solutions to the problem of mammalian exposure to AAs. We focused on the genes that expanded in *Pac. aristolochiae* and upregulated in AAI‐treated larvae, which may play a critical role in antioxidant defense and AAI detoxification. The gene set yielded 35 genes, including four *PGRs*, as well as genes belonging to the *SLC22*, *CYP3* and lipase gene families (Table S3), and we further identified the human homologs of these *Pac. aristolochiae* genes by homology search. Among the 35 genes, we found only one *PGR* gene that displayed a reciprocal best hit to *PTGR1* in the human genome (Figure 6A), whereas other *Pac. aristolochiae* genes exhibited low sequence similarity when compared to their best hits and were not the top matches in reverse comparisons (Figure 6B; and Table S3), suggesting large sequence divergence from human genes. Further comparisons showed that both sequence and structural similarity between *ParPGRs* and human *PTGR1* even surpasses that between *PTGR1* and its paralogs (*PTGR2* and *PTGR3*) within humans (Figures S23 and S24), indicating conserved enzyme activity and function between insects and humans. *PTGR1* is a bifunctional enzyme involved in the metabolism of 15‐oxo‐prostaglandins and leukotriene B4, which can effectively reduce cytotoxic lipid peroxidation products and resist oxidative stress [39, 43]. The functional description of *PTGR1* naturally highlights the mechanisms of AAI toxicity that have been revealed in mammals, particularly oxidative stress‐mediated mitochondrial dysfunction. Therefore, we further investigated whether *PTGR1* might play a role in promoting mammalian AA resistance. We first evaluated the expression profiles of *PTGR1* in the human kidney, one of the major target organs of AA, based on the single‐cell atlas [44]. The single‐cell dataset of the Kidney Precision Medicine Project (KPMP) comprised 225 177 high‐quality cells derived from the healthy reference, acute kidney injury (AKI) and chronic kidney disease (CKD) samples [44] (Figure 6C,D). The kidney cells were divided into 12 distant cell clusters belonging to the epithelial, endothelial, interstitial, and immune cells, which can be further sub‐classified into 54 subpopulations. Among them, the cell clusters of the most abundant epithelial cells were spatially related to different segments of nephrons (Figure 6C). The *PTGR1* gene exhibited a cell type specific expression pattern in the kidney and was also associated with disease status. Specifically, *PTGR1* was highly expressed in kidney epithelial cells and was the marker gene of the cell clusters of intercalated cells (IC), principal cells (PC) and proximal tubule (PT) (Figure 6E,F, Wilcoxon rank sum test, adjusted *p* < 0.001). In addition, in the principal cells, *PTGR1* expression levels in AKI samples were significantly lower than those in healthy reference or CKD samples (Wald test adjusted *p* < 0.01, Figure 6F). We subsequently conducted differential analysis at a higher resolution to identify potentially affected cell subpopulations, revealing that four cell subclasses including the proximal tubule segment 3 epithelial cells (PT‐S3), connecting tubule epithelial cells (CNT), connecting tubule intercalated cell type A (CNT‐IC‐A) and connecting tubule principal cells (CNT‐PC) showed AKI‐associated low‐expression pattern of *PTGR1* (Figure 6G,H). Similarly, analysis of the KPMP single‐nucleus dataset also supported that *PTGR1* was down‐regulated in PT cells of AKI samples, especially in the degenerative PT (dPT) cells (Figure S25), suggesting that the down‐regulation of *PTGR1* may be associated with oxidative stress and injury of PT and other renal tubular epithelial cells during AKI. Besides, in contrast to AKI, an opposite trend of gene expression changes was observed in chronic disease conditions, with significant upregulation of *PTGR1* in CKD samples in the IC and thick ascending limb (TAL) cells (Wald test adjusted *p* < 0.05, Figure 6F,G), suggesting potential compensatory mechanisms in chronic pathogenesis.

To investigate *PTGR1*’s protective role against AAI‐induced epithelial injury, we conducted functional studies in the human bladder epithelial cell line RT4, which exhibits high sensitivity to AAI similar to renal tubular epithelial cells [45]. First, *PTGR1* expression was knocked down using RNA interference. Quantitative PCR confirmed a significant reduction of *PTGR1* mRNA levels (*T*‐Test *p* < 0.001, Figure 6I). *PTGR1* knockdown cells showed markedly increased susceptibility to AAI‐induced cytotoxicity (Wilcoxon rank sum test *p* < 0.05, Figure 6J) and elevated production of 4‐hydroxy‐2‐nonenal (4‐HNE), a specific marker of oxidative stress and lipid peroxidation [46], as demonstrated by western blot analysis (*T* ‐Test *p* < 0.05, Figure 6K; Figure S26). Conversely, *PTGR1* overexpression achieved by plasmid transfection resulted in approximately nine‐fold upregulation of *PTGR1* expression (*T* ‐Test *p* < 0.001, Figure 6L). These *PTGR1*‐overexpressing cells exhibited significantly improved viability following AAI treatment (Wilcoxon rank sum test *p* < 0.01, Figure 6M) and reduced 4‐HNE accumulation (*T* ‐Test *p* < 0.05, Figure 6N; Figure S27). These complementary genetic approaches consistently demonstrate that *PTGR1* confers protection against AAI‐induced cytotoxicity in epithelial cells by mitigating oxidative stress. Our findings highlight *PTGR1* as a potential therapeutic target for AA‐related diseases including AAN.

## 3 Discussion

In this study, we investigated the adaptive strategies of *Pac. aristolochiae* to cope with highly mutagenic and nephrotoxic AAs, which induce oxidative stress, mitochondrial damage, and DNA adduct formation in mammals. Our findings suggest that AAs may be detoxified through biotransformation into low‐toxicity derivative metabolites (e.g. AAIa) and resisted by the butterfly's robust antioxidant capacity. Meanwhile, since biotransformation of AAs can also lead to DNA adduct formation—the basis of their carcinogenic effects in humans—we investigated how these butterflies avoid DNA damage and maintain genome stability. The results imply a dual strategy: restricted distribution of AAs in developmental and reproductive tissues, coupled with low metabolic activation in wing discs and selective barriers in germ cells, and enhanced DNA repair mechanisms in other vital tissues. Importantly, the AA‐induced antioxidant strategies in butterflies may provide potential therapeutic targets for AA‐associated human diseases, such as AAN.

On a molecular scale, the above findings highlight the importance of preventing DNA damage in critical organs that are most responsible for sustaining life activity and heritable genetic material, providing insights into the results of the evolutionary arms race between detoxification in *Pac. aristolochiae* butterflies and defense in Aristolochiaceae plants that has been ongoing for tens of millions of years. On a broad scale, there are over 200 000 lepidopteran species, and these butterflies and moths feed on a wide variety of host plants and have acquired diverse abilities to defuse host defenses and even utilize plant toxins. Our study provides a prime example of the translational potential of insect research, which may deepen understanding of insect responses to plant toxins and protective mechanisms and thus lead to clinical solutions for humans. Currently, AA‐associated health problems are still reported worldwide due to their environmental pervasiveness and inadequate restrictions on their use. Advances in genome profiling have expanded the list of AA‐associated cancers, including urothelial cancers, liver cancer, and renal cell carcinoma. Thus, diseases associated with AAs continue to pose a public health risk that requires global action [47]. Elucidating the mechanisms underlying how butterflies resist AA toxicity will undoubtedly provide insights into the prevention, monitoring, and treatment of AA‐related diseases in humans.

Clarifying the metabolism of AAs in phytophagous butterflies is an intriguing and valuable research question in itself, as the balance between the detoxification and bioactivation of AAs determines their effects—a topic that has been thoroughly studied in humans since the beginning of this century. Our study identified multiple AA metabolites in *Pac. aristolochiae* following exogenous AAI treatment. The detection of the low‐toxicity derivative AAIa supports the occurrence of metabolic detoxification, whereas the presence of ALI suggests a potential metabolic activation pathway similar to that observed in mammals. To dissect these mechanisms further, we inferred and characterized candidate enzymes involved in AAI metabolism, providing new insights previously missing from insect studies. Among the first‐phase enzymes, the *CYP3* clan and its member *CYP337* were expanded in *Pac. aristolochiae* and upregulated after AAI treatment (Figures 2E and 3B). *CYP337* is an environmental response gene in herbivorous insects and is involved in the metabolism of toxic chemicals [48], suggesting its potential role in AA detoxification in butterflies. Moreover, *NQO1* has been identified as the most efficient nitroreductase activating AAI in mammals [31]. However, the absence of *NQO1* homologs in butterflies led us to speculate that other reductases, such as the *SDR* and *AKR* families identified in this study, may play a role in AAI metabolism (Figure 3A,B). Inspired by these findings, we confirmed that human *AKR1A1*, a homolog of butterfly *AKRs*, is also involved in AAI bioactivation [49], providing a reference for further elucidating the mechanism of AAI‐induced carcinogenesis. Similarly, some phase II enzymes (e.g., *UGTs*) revealed in this study may also be involved in AA detoxification. Taken together, our findings indicate that these enzymes are likely responsible for the metabolism of AAs to confer resistance to these toxins, shedding light on the mechanisms of toxicity and also offering potential strategies for risk assessment and prevention in humans.

It is noteworthy that, although we demonstrated the formation of DNA adducts throughout the life cycle of *Pac. aristolochiae*, these adducts were absent in critical developmental and reproductive organs, such as embryos, wing discs, and testes. This protection might be achieved through multiple mechanisms, including physical restriction of AA entry, low expression of bioactivating enzymes and high expression of DNA repair‐associated genes. While the majority of AA intake occurs during the larval feeding period, the highest levels of DNA adducts are observed in the adult stage, exceeding 1800 adducts per 10^8^ nucleotides—a value substantially higher than that detected in human kidney tissue (<400 adducts per 10^8^ nucleotides) [11, 50]. This strong mutagenic pressure implies that a generalized mechanism in adult organs is necessary to avoid DNA damage and maintain physiological function. Although DNA repair‐associated gene families were not found to be expanded in the comparative genomic studies, transcriptomic analysis showed that genes involved in the FA and NER pathways are much higher in species that feed on Aristolochiaceae than in other species (Figure 4G,H), which may ensure the rapid and accurate repair of bulky DNA adducts, thereby improving overall repair efficiency. Interestingly, a recent study on long‐lived bowhead whales revealed that amino acid substitutions coupled with high expression levels of the DNA repair gene CIRBP enhance the repair capacity, demonstrating the value of non‐model organisms [51]. Similarly, exploring the unique adaptation in butterflies may offer insights for human medical research. Understanding the repair mechanism of AA‐induced DNA lesions can inform the development of new therapeutic strategies, such as enhancing repair pathways to resist environmental mutagens or sensitizing cancer cells to treatments.

In addition to their carcinogenic effects, AAs pose a significant risk of kidney injury in humans, primarily through oxidative stress‐mediated mitochondrial dysfunction, leading to tubular epithelial cell apoptosis and necrosis [10]. Current clinical management of AAN relies on symptomatic therapies (e.g., glucocorticoids, anti‐fibrotic agents, and immune‐modulators) that fail to address the root cause of AA‐induced toxicity. Although some antioxidants (including *N‐*acetylcysteine, vitamin E, and some natural bioactive compounds) show protective potential in experimental systems [21, 22, 23], their non‐specific mechanisms limit clinical translation. Our evolutionary‐guided approach using Aristolochiaceae‐adapted butterflies provides unique insights, as these insects have developed specialized resistance through prolonged coevolution with Aristolochiaceae plants. Despite the discovery of conventional antioxidants likely providing general protection rather than AA‐specific defense, e.g., expanded gene families and DEGs involved in oxidative stress response such as catalases, our results revealed that the *PGR* family showed both significant gene expansion and marked upregulation upon AAI exposure in *Pac. aristolochiae*, indicating strong selective pressure for AA‐specific detoxification. Coincidently, our meta‐analysis of human single‐cell atlases demonstrated the expression of *PTGR1*, a human homolog of *PGR*s, was associated with kidney injury. And the functional validation in epithelial cells further confirmed *PTGR1*’s protective role: overexpression reduced AAI‐induced cytotoxicity and 4‐HNE, a lipid peroxidation product and a critical mediator of oxidative stress, while knockdown exacerbated damage (Figure 6). As an oxidoreductase detoxifying lipid peroxidation products like 4‐HNE, *PTGR1*’s protective effects likely represent a more general component of cellular defense against oxidative stress—extending beyond AA‐specific bioactivation to diverse environmental toxins (e.g., other phytotoxins such as terpenes or cardiac glycosides that induce ROS). Although detailed mechanisms require further elucidation, these findings position *PTGR1* as a promising and valuable molecular target for developing specific interventions against AA and related oxidative toxicities.

Taken together, our findings provide insights into the mechanisms by which herbivorous insects tolerate toxic plant compounds such as AAs, highlighting the value of long‐term systematic studies using herbivorous insects as models. By integrating multidisciplinary approaches, we propose potential detoxification and defense strategies that may inform the development of therapeutic interventions for AA‐associated human diseases. These nature‐ and evolution‐based explorations underscore the potential of leveraging ecological adaptations to address human health challenges caused by phytotoxic substances.

## 4 Methods

### 4.1 Chemicals and Enzymes

Aristolochic acid I (AAI, CAS No. 313‐67‐7) and aristolochic acid II (AAII, CAS No. 475‐80‐9) were obtained from Shanghai Standard Technology Co., Ltd. (Shanghai, China). 2′‐deoxyadenosine (dA) and 2′‐deoxyguanosine (dG) were purchased from Alfa Aesar Corp. (Ward Hill, MA, USA). ^15^N_5_‐dA and ^15^N_5_‐dG were purchased from Cambridge Isotope Laboratories, Inc. (Andover, MA, USA). DNase I (deoxyribonuclease I from bovine pancreas, Type IV), nuclease P1 (from *Penicillium citrinum*), alkaline phosphatase (from *E. coli*), and phosphodiesterase I (from *Crotalus adamanteus* venom) were purchased from Sigma‐Aldrich (St. Louis, MO, USA). LC‐MS grade formic acid and acetonitrile were acquired from Sigma‐Aldrich (St. Louis, MO, USA) and Fisher Scientific Corp. (Waltham, MA, USA), respectively. Water was produced from a Millipore ultrapure water system (Burlington, MA, USA).

### 4.2 Insect Feeding, Treatment, and Sample Preparation

All insects used in this study were obtained from Shanghai Zoological Park. Larvae (first or second instars) and adult females were collected, reared in 0.5 × 0.4 × 0.5 m cages at room temperature, and fed with host plants (for larvae) or honey water (for adults). The laid eggs were used in studies or hatched into larvae and reared into pupae and adults for tests in studies. Sample collection spanned four years.

### 4.3 Sampling for Nanopore Sequencing, Hi‐C Analysis, and RNA Sequencing

For nanopore sequencing and Hi‐C library construction, high‐molecular‐weight genomic DNA was independently isolated from a fifth instar larva of *Pac. aristolochiae* and from a fifth instar larva of *Pap. polytes*. Additionally, four samples from four developmental stages (egg, larva, pupa and adult) of each species were used to extract total RNA for gene prediction during genome annotation.

### 4.4 Sampling for Absorption, Distribution, Metabolism, Elimination (ADME) Assay, and Transcriptomic Study on Larvae with or without AAI Treatment

The fifth‐instar larvae of *Pac. aristolochiae* and *Pap. polytes* were reared in the petri dishes (with three individuals per group). The AAI‐treated groups were fed *Aristolochia debilis* or *Citrus* sp. leaves, the surfaces of which were sprayed with 1 mg/mL AAI solution. The control groups were fed normal leaves without exogenous AAI treatment. After 18 h of treatment, the leaves were withdrawn and weighed to estimate food consumption and AAI intake. After another 6 h, when no more feces were excreted, the feces and osmotic fluid were collected, and the larvae were dissected. The alimentary canal and osmeterium were removed (Figure S2), and the remaining bodies were flash‐frozen in liquid nitrogen for subsequent analysis of AAI metabolites and RNA isolation. The samples were stored at −80°C until use.

### 4.5 Sampling of the Embryos for DNA Adducts Detection in *Pac. aristolochiae*

The newly laid fertilized eggs and middle‐stage eggs, collected 3–5 days after oviposition, were pooled and used for DNA isolation without any prior processing. Figure S4A illustrates the embryo morphology at 72 h after oviposition. For the latest embryonic stage, larvae were dissected from the eggs just before hatching (Figure S4B). Since newly hatched first instar larvae consume their eggshells upon emergence, any AAs present on the eggshell surface could be ingested. To assess DNA adduct levels specific to embryonic development and exclude the influence of post‐hatch ingestion, the eggshells were washed to remove all of the yellow coating material deposited during oviposition, which may contain AAs. Subsequent experiments were then conducted using the newly hatched larvae. Figure S4C,D displays the egg before and after washing, respectively, while Figure S4E portrays the newly hatched first instar larvae.

### 4.6 Sampling of Pupae and Pupal Wings for DNA Adducts Detection, AAI Content Determination and RNA‐seq Analysis in *Pac. aristolochiae*

For the detection of DNA adducts, whole pupae (Figure S5A) and the pupal wings dissected from additional pupae (Figure S5B) were utilized. As for determining AAI content and conducting RNA‐seq analysis, another set of pupae was used. These pupae were halved from the center into bilateral parts (Figure S5C), one part was used for determining AAI content, and the other part was used for RNA‐seq analysis. The dissected unilateral wings and the remaining halves of the pupae were separately flash‐frozen in liquid nitrogen for subsequent analysis.

### 4.7 Sampling of Testes for DNA Adducts Detection, Spatial Transcriptomic Analysis and Fluorescence Imaging of Alkyne‐Tagged AAI in *Pac. aristolochiae*

For the detection of DNA adducts, adults (Figure S19A) with legs and wings removed, as well as testes isolated from another set of adults (Figure S19B), were flash‐frozen in liquid nitrogen for subsequent analysis. For spatial transcriptomic analysis, the whole testis and thorax tissue were dissected from adults, washed and placed in embedding cassettes that were pre‐coated with OCT. The cassettes were then placed in dry ice and transferred to −80°C until ready for cutting. In the fluorescence imaging experiment, adults were treated with alkyne‐tagged AAI (Figure S19C), 16 h after treatment, the whole testis and thorax tissue were dissected and embedded in OCT following the procedures mentioned above.

### 4.8 Qualitative Profiling and Relative Quantitative Analysis of AAI and Its Metabolites

The larval bodies, feces and osmotic fluid were homogenized in saline. The mixtures were spun at 14 000 × *g* for 10 min, and then the supernatants were mixed with a one‐half volume of methanol and spun again at 14 000 × *g* for 5 min to remove precipitated proteins. Aliquots of the final supernatants were analyzed to quantify the levels of AAI and its metabolites. Qualitative profiling was performed on an Agilent 1290 Infinity ultrahigh‐performance liquid chromatography (UHPLC) system (Santa Clara, California, USA) connected to an SCIEX Triple time‐of‐flight (TOF) 4600 mass spectrometer (Framingham, Massachusetts, USA) with reference to the Agilent‐Nature Standard Natural Product Personal Compound Database and Library (PCDL) and reported data. Analyses of the samples were performed on an Agilent ZORBAX RRHD SB‐C18 column (100 × 2.1 mm, 1.8 µm) with mobile phase A and B being water and acetonitrile (both contained 0.1% (v/v) formic acid), respectively, at a flow rate of 0.3 mL/min and the column temperature of 25°C. The gradient program was linear, starting at 2 min from 10% mobile phase B to 20% over 3 min, at 5 min from 20% to 30% over 6 min, at 15 min from 30% to 45% over 1 min, at 29 min from 45% to 95% over 4 min, and at 34 min from 95% to 10% over 1.1 min and stopped at 38 min. The mass spectrometer was run under the positive electrospray ionization (ESI^+^) mode with the mass range being 50–1700. The mass spectrometric parameters for mass spectral analyses were as follows: Ion Source Gas 1, 50; Ion Source Gas 2, 50; Curtain Gas, 35; Ion Spray Voltage Floating, 5000 V; Ion Source Temperature, 500°C; Declustering Potential, 100; Collision Energy, 10. The instrumental parameters for MS/MS analyses were as follows: the mass range, 50–1700; Declustering Potential, 100; Collision Energy, 40; Collision Energy Spread, 20; Ion Release Delay, 30; Ion Release Width, 15.

The metabolites were identified with different confidence levels on the basis of their accurate masses and diagnostic fragments, and also their retention time (t_R_) when the reference standards were available (Figure S3 and Table S1). The amounts of these metabolites were estimated according to the intensities of their parent ions [M+H]^+^ or [M+H‐H_2_O]^+^) normalized against the weights of the samples.

### 4.9 Quantitative Analysis of AAI/II‐DNA Adducts

The formation of DNA adducts in distinct developmental stages of *Pac. aristolochiae* was quantitatively estimated. Middle‐ and latest‐stage embryos, newly hatched first‐instar larvae, fifth‐instar larvae, whole pupae and adult bodies (without wings or legs) were collected. The formation of DNA adducts in pupal wings and adult testes was also determined through independent experiments to study DNA lesions in reproductive and developmental organs, respectively. The samples were flash‐frozen in liquid nitrogen and then immediately homogenized in 50 mm Tris‐HCl (pH 8.0) containing 10 mm EDTA (TE buffer). DNA was extracted from the homogenates via the phenol‐chloroform method and then enzymatically digested following previously described procedures [50].

The formation of DNA adducts by the major AA analogs AAI and AAII was determined. dA‐/dG‐ALI, dA‐/dG‐ALII, ^15^N_5_‐dA‐/dG‐AAI, and ^15^N_5_‐dA‐/dG‐AAII standards were synthesized, and the DNA adducts were measured through isotope‐dilution LC‐MS/MS quantitation as previously described [52]. The limits of detection (LODs) were determined as follows: 3, 7, 2, and 3 adducts per 10⁸ nucleotides for dA‐AAI, dG‐AAI, dA‐ALII, and dG‐ALII, respectively, given an input of 10 µg of DNA. To ensure reliable detection across tissues with varying adduct loads, we first estimated typical adduct levels and DNA yields in pilot experiments. Based on these pilot data, tissues with anticipated low adduct abundance (e.g., eggs and testes) required pooling of multiple individual samples to obtain a total DNA mass sufficient for the assay's sensitivity. The input DNA amount for each sample was provided in the raw data table.

### 4.10 Synthesis of Alkyne‐Tagged AAI

Aristolochic acid IVa (10.0 mg, 0.028 mmol), 3‐bromopropyne (10.0 mg, 0.084 mmol), and anhydrous potassium carbonate (13.3 mg, 0.112 mmol) were dissolved in DMF and stirred at 40°C for 3 h, followed by adding sodium hydroxide solution (1.5 M, 0.1 mL) and stirring at 40°C for 3 h. The reaction solution was allowed to cool down to room temperature, poured into a large amount of water, and acidified to pH = 1 with hydrochloric acid. The resulting solid mixture was collected by filtration and submitted to silica gel thin layer chromatography for purification, which afforded the target compound (5.8 mg, 52.4% in yield) as a yellow solid powder.

### 4.11 Click Chemistry‐Based Fluorescence Imaging for In Situ Detection

To track the cellular localization of monoalkyne AAI, fluorescence imaging studies were designed and conducted using TAMRA azide, 5‐isomer, an orange‐emitting fluorescent dye (Abcam). Whole testis and thorax tissue samples from adult *Pac. aristolochiae* were frozen and embedded in optimal cutting temperature (OCT) compound. Sections of 150–200 µm thickness were obtained using a cryostat microtome (CRYOSTAR NX50). The tissue sections were incubated with click chemistry reaction buffer (5 mm CuSO_4_, 50 mm ascorbic acid, and 1 µm TAMRA azide in PBS) for 30 min in the dark at room temperature. Following the click chemistry reaction, the sections were washed three times with PBS and subsequently stained with DAPI for nuclear counterstaining for an additional 10 min. The fluorescence signals were measured using an inverted fluorescence microscope (Nikon Eclipse C1). Images of the intracellular uptake of the monoalkyne AAI and DAPI were obtained using the Cy3 channel (excitation 510–560 nm and emission 590 nm) and the DAPI channel (excitation 330–380 nm and emission 420 nm), respectively. The specificity of the click chemistry reaction for imaging the intracellular delivery of the monoalkyne AAI was confirmed using negative control samples. Specifically, one control section was incubated with click chemistry reaction buffer without TAMRA azide to confirm the absence of interference from spontaneous tissue fluorescence. Another control section was obtained from untreated mouse heart tissue to ensure that no signal interference was caused by residual fluorescent probes or nonspecific binding, following the same procedure described above.

### 4.12 Karyotyping

Chromosome slides were prepared using the air drying technique. Gonads without fat and membranes were removed into a 0.005% fresh colchicine‐hypotonic solution on a slide for 1 h at room temperature. After hypotonic treatment, the organs were transferred to 2–3 drops of freshly prepared fixative I (acetic acid 3 mL/ethanol 3 mL/double‐distilled water 4 mL) for 1 min. Dissect the organ quickly to spread the cells and cell mass outwards. During the spread process, drop fresh fixative II (acetic acid 1 mL/ethanol 1 mL) on the whole slide when the fixative I spreads to the slide margins. After a few minutes, when the slides were dry, add 2 drops of acetic acid. Leave the slides at room temperature for 1 day to dry completely. Stained the slides with 10% Giemsa solution for 15 min at room temperature. After staining, each slide was washed with running water and dried. Images of chromosomes were captured using a 100× oil immersion objective (OLYMPUS, IX73).

### 4.13 Genome Assembly, Annotation, and Comparative Analyses

Genomic DNA was extracted from the fifth‐instar larvae of *Pac. aristolochiae* and *Pap. polytes* using the CTAB‐based method and was used for Oxford Nanopore and Hi‐C sequencing at Grandomics (Wuhan, China). High‐molecular‐weight DNA was extracted and purified via a Genomic‐tips Kit (Qiagen, Germany). For the construction of the Nanopore sequencing library, ligation kits were used to repair DNA damage, and a polyA tail was added to connect the adapter containing the motor protein. Finally, a PromethION sequencer was used to conduct single‐molecule real‐time DNA sequencing to obtain the original sequencing data. Base calling was first performed to convert the raw FAST5 files to FASTQ format with Guppy v3.7 (https://nanoporetech.com/document/Guppy‐protocol). The raw reads with mean quality scores less than seven were then filtered. NextDenovo [53] was used for correcting the raw reads of the Oxford Nanopore data, and SMARTdenovo [54] was used for the *de novo* assembly of *Pac. aristolochiae* and *Pap. polytes* genomes. Using Nextpolish [55], the sequences were then polished three and four times based on the Nanopore long reads and Illumina short reads, respectively. The Hi‐C data were processed using the Hic‐Pro pipeline [56], and LACHESIS [57] was used for chromosome scaffolding according to the read pairs with valid interactions. The completeness of the assembly was evaluated using BUSCO v3.0.2 [58] on the basis of the conserved single‐copy gene set “insecta_odb10” [59].

For repeat annotation, tandem repeats were identified using TRF v4.07 [60]. MITE‐hunter [61], LTR_retriver v2.0 [62] and RepeatModel v1.0.11 [63] were employed to generate the species‐specific repeat library, and then RepeatMasker v1.331 (http://www.repeatmasker.org) was used for genome‐wide scanning of the repeat sequences.

Augustus v3.3.1 [64] and GeMoMa v1.6.1 [65] were used for ab initio and homology‐based prediction of the protein‐coding genes. For the latter methods, gene models from *D. melanogaster* [66], *Bombyx mori* [67] (GCF_014905235.1), *Danaus plexippus* [68] (GCF_009731565.1), *Papilio glaucus* [69] (GCA_000931545.1), *Papilio machaon* [70] (GCF_912999745.1), and *Papilio xuthus* [71] (GCF_000836235.1) were used. The RNA‐seq data from different development stages of *Pac. aristolochiae* and *Pap. polytes* were aligned to the reference genome assemblies of the corresponding species using HISAT2 v2.1.0 [72]. The transcripts were assembled using StringTie v1.3.4 [73], and the coding regions were predicted using PASA v2.3.3 [74]. EVM v1.1.1 [75] was used to integrate gene structures from different methods, with weights set to 10, 5, and 2 for transcriptome‐based, homology‐based, and ab initio predictions, respectively.

Protein functions were predicted on the basis of homology inference. The Gene Ontology and protein family entries were determined via search against the InterPro database [76] using InterProScan v5.46 [77], and the KEGG pathways were annotated with reference to the eggNOG database using eggNOG‐mapper v2.1.9 [78]. The “buildGOmap” function of the clusterProfiler package [79] was used to map the high‐level GO terms, and functional enrichment of focal gene sets was performed using the “enricher” function. The *p* values were calculated on the basis of the hypergeometric distribution, and the “FDR” method was applied for multiple‐testing correction.

For the comparative genomics study, in addition to the assembled and annotated genomic data of *Pac. aristolochiae* and *Pap. polytes*, the protein coding sequences of *D. melanogaster* [66], *Plutella xylostella* [80], *B. mori* [67], *Manduca sexta* [81], *Heliconius melpomene* [82], *Pieris rapae* [83], *Sericinus montelus* [84], *Pap. glaucus* [69], *Pap. machaon* [70] and *Pap. xuthus* [71] were downloaded. Only the longest transcripts were used for downstream analyses. The synteny between the genomes of *Pac. aristolochiae*, *S. montelus* and *Pap. polytes* were analyzed and visualized using the JCVI pipeline [85]. The orthogroups of homologous genes were identified using OrthoFinder v2.5.4 [86]. The species phylogeny was reconstructed on the basis of the protein sequence alignments of all single‐copy orthologous genes. The R8s algorithm [87] was used to estimate the species divergence time with four calibration points: the root node (286 Mya), the common ancestor of *B. mori*, *M. sexta* and eight butterfly species (56–140 Mya), the common ancestor of eight butterfly species (69–119 Mya), and the common ancestor of *Pap. polytes*, *Pap. machaon*, and *Pap. xuthus* (16–40 Mya), which were acquired from the TimeTree database [88] (http://www.timetree.org/).

Changes in gene family sizes were analyzed using CAFE v4.2.1 [89] on the basis of the numbers of genes in orthogroups and the time‐calibrated phylogenetic tree. Orthogroups with branch‐specific *p* values less than 0.01 (calculated based on the Viterbi method) were identified as significantly expanded or contracted gene families.

For phylogenetic analyses of gene families, protein sequences were aligned using MUSCLE v3.8 [90]. A maximum likelihood tree was reconstructed using RAxML v8 [91] with the “PROTGAMMAJTT” amino acid substitution model, and the bootstrap number was set to 100. The phylogenetic tree was visualized using iTOL v6 [92], and gene positions and structures were displayed using the gggenes package (https://wilkox.org/gggenes/).

To evaluate gene selection pressures, the translated alignment mode in PRANK [93] was used to align the CDSs of each gene pair, and KaKs Calculator 2.0 software [94] was used to estimate the Ka/Ks value via the “LWL” method.

To identify the homologs of *Pac. aristolochiae* genes in humans, the human protein sequences (version GRCh38.p13) were downloaded from https://www.gencodegenes.org/human/release_38.html. Bidirectional BLASTP comparisons revealed 5611 reciprocal best‐hit gene pairs, representing the conserved homologous between two genomes.

### 4.14 Bulk Transcriptomic Analyses

Fifth instar larvae of *Pac. aristolochiae* treated with or without AAI were subjected to RNA sequencing. At the pupal stage, wing discs were dissected from two males and two females at 5–6 days after pupation, together with the remaining pupae, for RNA‐seq. Total RNA was extracted using the RNeasy Plus Mini Kit (Qiagen, Germany). After quality control for RNA purity and integrity, cDNA libraries were constructed using the TruSeq RNA Library Prep Kit (Illumina) and sequenced on the NovaSeq 6000 platform (Illumina) at Grandomics (Wuhan, China).

For quality control, low‐quality bases were trimmed via Trimmomatic v0.39 [95] with the recommended parameters. The filtered RNA‐seq data were then aligned to the reference genome of *Pac. aristolochiae* using STAR v2.7.5 [96] with the parameters “–sjdbOverhang 149, –alignIntronMin 20, –alignIntronMax 100 000, –outSAMmultNmax 1, –outSAMmapqUnique 60”, and the gene expression levels were quantified using RSEM [97] according to the alignment results. Sample information is shown in Table S2.

Raw counts were normalized using DESeq2 [98], and the Wald test was used to calculate the significance of gene expression differences between groups. Genes with adjusted *p* values less than 0.05 and fold changes greater than 2 were identified as differentially expressed. For principal component analysis, the counts were transformed using the variance stabilizing transformation (vst), and the first two principal components were used for visualization. For the expression patterns of target genes, the log‐transformed FPKM values (log_2_(FPKM+1)) were scaled across samples, and heatmaps were created with the pheatmap package (https://cran.r‐project.org/web/packages/pheatmap/index.html).

For cross‐species comparisons, the transcriptome data of *Pap. polytes*, *Pap. machaon*, and *Pap. xuthus* were downloaded from the SRA database (accession numbers PRJNA634605 [99], PRJNA728224 [100], PRJNA270386 [101], PRJNA270384 [102], and PRJNA718996 [102]. The data were then aligned to the reference genomes of *Pap. polytes*, *Pap. machaon* [65] (GCF_912999745.1), or *Pap. xuthus* [71] (GCF_000836235.1), and gene expression levels were quantified as previously described. The orthologous gene pairs of *Pac. aristolochiae* and *Pap. polytes* (9669 genes), i.e., reciprocal BLASTP best hits, and the single‐copy gene families in four Papilionidae species (7368 genes) were used for the downstream network analyses. Gene coexpression networks were constructed using the WGCNA algorithm [103]. Specifically, genes with low expression and mean FPKM values less than 2 were removed, and the remaining FPKM values were log‐transformed. Networks were then constructed using the “blockwiseModules” function with the parameters “networkType = “unsigned”, TOMType = “signed”, minModuleSize = 30, mergeCutHeight = 0.25”. The soft threshold was selected on the basis of the independence and connectivity of the network, which was set to 14 for the two‐species network and 12 for the four‐species network. Hub genes of each gene module were identified as those with Pearson correlations greater than 0.8 between the gene expression levels and the module eigengenes (the first principal component). Correlations between gene module eigengenes and biological traits, including species and tissues, were also calculated, and the *p* values for the correlations were computed using the “corP valueStudent” function.

### 4.15 Spatial Transcriptomic Analyses

One adult male *Pac. aristolochiae* was dissected, and the testes and remaining body without wings or legs were collected for spatial transcriptomic analysis using Stereo‐seq, which was performed in accordance with the standard protocol [104] at BGI Research (Shenzhen, China). Briefly, the samples were embedded in OCT and cryosectioned, and the tissue sections were placed on the surface of STOmics chips for fixation and permeabilization. The released mRNA molecules were captured in situ and reverse transcribed. The cDNA libraries were sequenced using the DNBSEQ platform. The raw spatial transcriptome data were processed using Stereo‐seq Analysis Workflow (SAW) v6 [105], and the adjacent 40 × 40 DNB bins were combined as one unit for downstream analyses. The count matrix with spatial coordinates was analyzed using the Seurat workflow [106]. Counts were normalized using the “SCTransform” method, and then PCA and UMAP were applied for dimension reduction. For cell clustering, the first 15 PCs were used to construct the nearest neighbor graph, and the Louvain algorithm was used to determine cell clusters with the parameter “resolution = 0.3”. The cell subsets belonging to the testis were extracted and reanalyzed, the PC numbers were set to 9, and the resolution was set to 0.2. The marker genes of each cell cluster were identified using the “FindAllMarkers” function with the parameters “test.use = “wilcox”, only.pos = TRUE”, and the top five or ten genes with the greatest fold changes were used for visualization via the “DoHeatmap” function.

### 4.16 Analysis of Human Kidney Single‐Cell Data

The single‐cell and single‐nucleus datasets of the human kidney were downloaded from https://atlas.kpmp.org/ with the doi numbers 10.48698/mgd0‐gz70 and 10.48698/mg7h‐bc51, respectively. The SeuratDisk package (https://github.com/mojaveazure/seurat‐disk) was used to convert the file format from h5Seurat to h5ad. The Scanpy workflow [107] was used for data processing based on the gene expression matrix and cell cluster annotations. The functions “pl.umap” and “pl.dotplot” were used for the visualization of cell and gene expression, and the function “tl.rank_genes_groups” was used for identifying the marker genes of each cell cluster or subpopulation with the parameters “method = wilcoxon, use_raw = False”. The pseudobulk method was used for the differential expression analysis between disease groups. Each cell cluster or subpopulation was analyzed separately. The summarized counts were calculated across all cells from one sample using the function “get_pseudobulk” of the decoupler package [108] with the parameters “layer = counts, mode = sum, min_cells = 50, min_counts = 1000”, and the raw counts were normalized using Deseq2 [98] with disease type as the design factor. Pairwise comparisons between healthy, AKI and CKD samples were performed using the Wald test.

### 4.17 Studies on the Human Homolog *PTGR1* in Human RT4 Cells

#### 4.17.1 Cell Culture and Drug Treatment

The RT4 cell line (*RRID: CVCL_0036*) was obtained from the Cell Bank of the Chinese Academy of Sciences (Shanghai, China). Cell identity was authenticated by short tandem repeat (STR) profiling at the National Collection of Authenticated Cell Culture. Additionally, the cells were tested and confirmed negative for mycoplasma contamination. RT4 cells were cultured in complete McCoy's 5A medium (BasalMedia, Shanghai, China) supplemented with 10% fetal bovine serum (Vivacell, Shanghai, China). All the cells were maintained in a humidified incubator at 37°C with 5% CO_2_.

#### 4.17.2 RNA Interference

*PTGR1* expression was silenced using small interfering RNA (siRNA). *PTGR1*‐targeting siRNA and negative control siRNA were purchased from IBSBIO (Shanghai, China). Transfections were performed using Lipofectamine 2000 (Invitrogen, Carlsbad, CA, USA) and McCoy's 5A medium (BasalMedia, Shanghai, China) according to the manufacturer's instructions. The sequences of the siRNAs used were as follows: control siRNA sense 5’‐UUCUCCGAACGUGUCACGUTT‐3’; control siRNA antisense 5’‐ ACGUGACACGUUCGGAGAATT‐3’; siRNA targeting PTGR1 (1) sense 5’‐ GCCAAAGUUGUGGAAAGUAUU‐3’; siRNA targeting PTGR1 (1) antisense 5’‐ UACUUUCCACAACUUUGGCUU‐3’; siRNA targeting PTGR1 (2) sense 5’‐ CAGAGAUUGUUAUCUAUCAGG‐3’; and siRNA targeting PTGR1 (2) antisense 5’‐ UGAUAGAUAACAAUCUCUGGG‐3’.

#### 4.17.3 Gene Overexpression

*PTGR1* overexpression vector (pcDNA3.1‐3xFlag‐PTGR1) and empty vector (pcDNA3.1) were purchased from NewHelix Biotech (Shanghai, China). For *PTGR1* overexpression, RT4 cells were transfected with 2.5 µg of *PTGR1* plasmid or empty vector using Lipofectamine 3000 (Invitrogen, Carlsbad, CA, USA) according to the manufacturer's protocol.

#### 4.17.4 Cell Proliferation Assay (CCK‐8)

RT4 cells were seeded in 96‐well plates and cultured overnight in a 5% CO_2_ incubator at 37°C. The culture was continued with medium containing a final concentration of 0.125, 0.25, 0.5, 0.75, 1, 1.5, or 2 µm AAI or an equivalent volume of DMSO. After 24 h, the culture medium was replaced with CCK‐8 reagent (Dojindo, Kumamoto, Japan), and the plates were incubated at 37°C for 1 h. The absorbance at 450 nm was measured using a microplate reader. The relative cell proliferation rate was calculated as: (OD_experiment_ − OD_blank_)/(OD_control_ − OD_blank_) × 100%. DMSO‐treated cells were used as controls.

#### 4.17.5 Western Blot for 4‐HNE‐Protein Adducts Production Determination

Total protein was extracted from RT4 cells using cell lysis buffer (Beyotime, China) containing 2% protease inhibitor cocktail (Beyotime, China) and 2% phosphatase inhibitor cocktail (Beyotime, China). The supernatants were collected after centrifugation at 10 000 × *g* for 20 min at 4°C, mixed with 20% loading buffer (Beyotime, China), and boiled at 100°C for 5 min. The protein concentration was measured using a BCA assay (Beyotime, China). Equal amounts of protein were separated on a 10% gradient gel and transferred onto 0.22 µm PVDF membranes (Millipore, USA). The membranes were blocked with 5% nonfat milk for 1 h and incubated with primary antibodies overnight at 4°C. After incubation with horseradish peroxidase (HRP)‐conjugated secondary antibodies (Beyotime, China) at room temperature for 2 h, the membranes were developed with Clarity Western ECL Substrate (Bio‐Rad, USA). All blots were imaged and quantified using an X‐ray machine (Bio‐Rad, USA), and the images were analyzed with Image Lab software. The following antibodies were used: β‐TUBULIN (Abmart, M30109s, 1:1000 dilution), 4‐hydroxynonenal (4‐HNE) (R&D Systems, MAB3249, 1:500 dilution), and PTGR1 (ABclonal, A4521).

#### 4.17.6 Quantitative Real‐Time PCR (qRT‐PCR) Assay

Total RNA was isolated using RNAiso Plus (Takara Bio, China), and cDNA was synthesized using HiScript III RT SuperMix for qRT‐PCR (Vazyme, China). qRT‐PCR was performed using ChamQ Universal SYBR qPCR Master Mix (Vazyme, China) according to the manufacturer's instructions. Each biological sample was run in technical triplicate, and average expression values were normalized against those of *GAPDH* using the 2^−^^ΔΔCT^ method. The sequences of the primers used were as follows: GAPDH forward, 5’‐ TCCTCCTGTGACCCTTTCGG‐3’; GAPDH reverse, 5’‐ GAAGGCGGCTGGGACTGC‐3’; PTGR1 forward, 5’‐GGAGCGAGATCCCTCCAAAAT‐3’; and PTGR1 reverse, 5’‐ GGCTGTTGTCATACTTCTCATGG‐3’.

### 4.18 Statistical Analysis

All statistical analyses were performed using R (version 4.1.0) and Python (version 3.12.8). For GO and KEGG enrichment analyses, the hypergeometric test was applied, followed by false discovery rate (FDR) correction for multiple testing. In the analysis of bulk RNA‐seq and single‐cell RNA‐seq pseudobulk data, the Wald test was used to assess differences between two groups based on the normalized counts, with FDR correction for multiple comparisons. For raw single‐cell RNA‐seq data, the Wilcoxon rank sum test was employed to identify cell cluster marker genes. The T‐test was applied to analyze qRT‐PCR and Western blot experimental results, while the Wilcoxon rank sum test was used for cell proliferation rate comparisons. All tests were two‐sided. No sample or data points were excluded from the analyses.

## Author Contributions

Y.L. was the lead contact and primary investigator, overseeing the project and securing funding, and also conducted a significant portion of the experiments. W.Z. significantly contributed to the research, provided critical input, and participated in project administration, supervision, and writing. L.M.S. also secured funding and contributed to the project supervision and writing. Y.B.Z.: Database retrieval, data analysis, bioinformatics analysis, and participated in writing. J.J.L.: Genome assembly, Hi‐C scaffolding, and gene annotation. J.Q.Z: Collection and identification of butterfly samples. Y.Y.L.: Human cell experiments. Y.S.H. and X.Y.Z.: DNA adducts detection. Z.Q.X. and T.P.X.: Qualitative and quantitative analysis of AAI and its metabolic products in insects. J.Z. and Y.Y.L.: Synthesis of monoalkyne AAI. J.J.S.: Quantitative analysis of AAI in pupae. Other authors participated in all aspects of the experiments. All the authors reviewed and edited the manuscript.

## Funding

This work was supported by the National Natural Science Foundation of China (grant nos. 32325009 and 32170420 to W.Z., 41991314 to Y.L.), and by grants from Benyuan Charity Young Investigator Exploration Fellowship in Life Science, The Feng Foundation of Biomedical Research, the Peking‐Tsinghua Center for Life Sciences, the Peking University Chengdu Academy for Advanced Interdisciplinary Biotechnologies, and the State Key Laboratory of Protein and Plant Gene Research to W.Z.

## Conflicts of Interest

The authors declare no conflicts of interest.

## Supporting information




**Supporting File**: advs73644‐sup‐0001‐SuppMat.docx.

## Data Availability

The Oxford Nanopore, Hi‐C sequencing, and whole‐genome sequencing data of *Pac. aristolochiae* and *Pap. polytes* were deposited in the NCBI Sequence Read Archive (SRA) database under the accession number PRJNA665105. And the RNA‐seq data of *Pac. aristolochiae* was under the accession numbers PRJNA665105 and PRJNA1179702. The single‐cell and single‐nucleus data of human kidney is available at the KPMP Data Atlas Explorer (https://atlas.kpmp.org/explorer/dataviz). The genome assemblies, gene annotations and the gene expression matrices of bulk RNA‐seq and spatial transcriptome data, and the raw data for quantitative analysis of AA metabolites, AA‐DNA adducts, cell proliferation, gene expressions, and 4‐HNE productions are available at the Figshare digital data repository with the DOI (https://doi.org/10.6084/m9.figshare.28052963). All scripts and codes are available at the Figshare digital data repository with the DOI (https://doi.org/10.6084/m9.figshare.28052963).
