# Supplementary material for: Mechanisms of Aristolochic Acid Resistance in Specialist Butterflies and Evolutionary Insights for Potential Protective Pathways
Source: Adv Sci (Weinh). 2026 Jan 4;13(19):e18072. doi: 10.1002/advs.202518072 (PMC13045329; doi:10.1002/advs.202518072)
Supplement: Supplementary file 1 — Supporting File: advs73644‐sup‐0001‐SuppMat.docx. [file ADVS-13-e18072-s001.docx]

Supporting Information

Mechanisms of aristolochic acid resistance in specialist butterflies and evolutionary insights for potential protective pathways

Yang Luan^1*^†, Yubo Zhang^2^†, Jingjing Li^3^, Jianqing Zhu^4^, Yuyang Lei^1^, Yushi Hu^1^, Zhenqiang Xin^5^, Tianpei Xie^5^, Jiang Zheng^6^, Yuanyuan Lin^6^, Jingjing Shen^7^, Yiyi Cao^1^, Xinyue You^1^, Jing Xi^1^, Jiaying Wu^1^, Weiying Liu^8^, Xinyu Zhang^1^, Yuanting Zheng^3^, David J. Lohman^9,10,11^, Leming Shi^3*^, Wei Zhang^2,12,13,14,15*^

**Supplementary Text**

**Expression patterns of *PGRs* in different Papilionidae butterflies**

*PGRs* showed significantly different expression patterns between copies and among species (Figure S28, one-way ANOVA, *P* < 2×10^−16^). In particular, *PGR1* genes were highly expressed and were not significantly different among the four species, suggesting that *PGR1* may play a conserved role with a constrained abundance. Notably, two new *PGR* copies were expressed at levels higher than or similar to *PGR1* in *Pac. aristolochiae*, whereas there was only one *PGR1* copy in other three *Papilio* species, indicating the expression of *PGR* genes was under precise regulation and may play a significant role in the detoxification of AAs in *Pac. aristolochiae*.

Figure S1. The photographs of butterfly samples used in the analyses.

The photographs of four developmental stages (egg, fifth instar larva, pupa, and adult) of *Pac. aristolochiae* (A) and *Pap. polytes* (B).

Figure S2. Sampling for ADME assay and transcriptomic study.

The fifth instar larvae of *Pac. aristolochiae* (A) and *Pap. polytes* (B) were housed in the petri dish, fed with the leaves of *Aristolochia debilis* (A) or citrus (B) that were mixed with 1 mg/mL AAI solution (C). *Pap. polytes* exhibited normal behaviors after 18 hours of AAI treatment. Dissection of *Pac. aristolochiae* (D) and *Pap. polytes* larvae (E, F) with the alimentary canal removed. Removing the osmeterium (G) and collecting the osmeterial fluid (H).


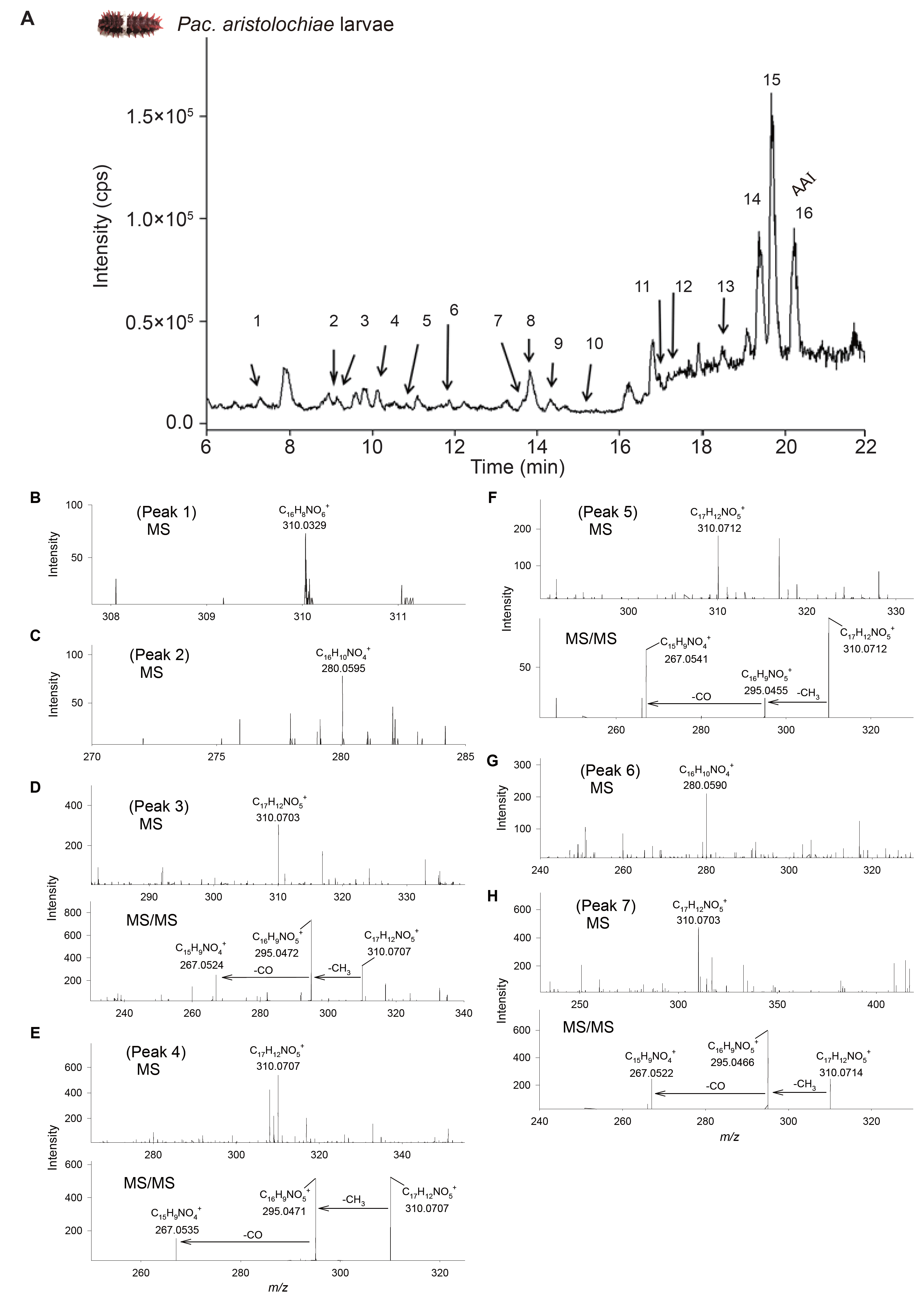


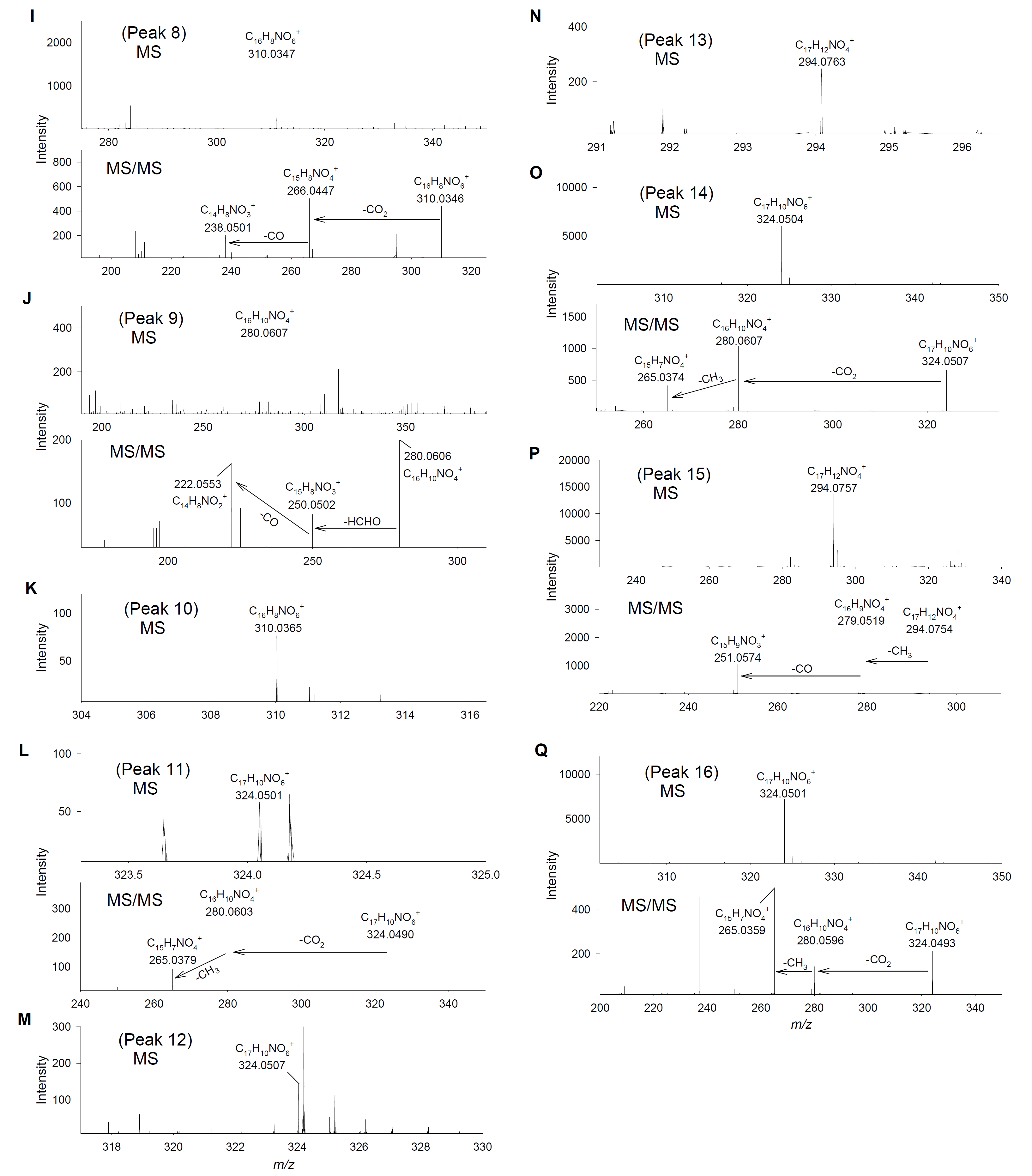


Figure S3. Selected ion chromatograms of feces sampled from *Pac. aristolochiae* larvae fed on *Aristolochia debilis* leaves coated with AAI solution and the corresponding mass spectra of the identified metabolites.

(A) AAI (16) and its possible metabolites are indicated as 1–15 in the figure (details in Table S1). The selected ion range was from *m/z* 279 to 325. (B-Q) MS and MS/MS spectra are shown for each metabolite (1–16), with precursor and fragment ion masses labeled in the figures.

Figure S4. Different development stages of the embryos.

(A) Middle stage embryos. (B) Late-stage embryos. (C) Eggs before washing. (D) Eggs after washing. (E) Newly hatched first instar larva consumes its eggshell.

Figure S5. Pupal wings dissection from *Pac. aristolochiae* pupae.

(A) Whole pupae. (B) A complete pupal wing dissected from a pupa. (C) Dissection of the pupal wings from pupae.

Figure S6. Chromosome-level reference genome of *Pac. aristolochiae* and *Pap. polytes*.

(A) The karyotype of *Pac. aristolochiae.* 29 pairs of chromosomes were determined by Giemsa staining. The heatmap shows the genome-wide chromatin interactions of *Pac. aristolochiae* (B) and *Pap. polytes* (C). The contigs were clustered into 29 and 31 chromosome groups, respectively, and ordered based on the Hi-C data. (D) Statistics of the draft and the chromosome-level genome assemblies. ‘S’ and ‘D’ represent single-copy and duplicated BUSCO scores, respectively.

Figure S7. Summary of the orthologous genes between *Pac. aristolochiae* and other Lepidoptera species.

The histogram shows the gene number in each species and the genes are classified according to the type of orthogroups. Singe copy means single-copy genes. Present in all species indicates other orthogroups present in all species with multiple gene copies. Lepidoptera, butterfly, and Papilionidae specific means orthogroups only existed in eleven Lepidoptera species, eight butterfly species, and six Papilionidae species, respectively. Species specific means species-specific orthogroups. Other means all other types. Unassigned means genes not belonging to any orthogroup.

Figure S8. Evolution of the *PGR*, *catalase* and *CYP337* genes in Papilionidae butterflies.

(A) Dot plot showing the synonymous substitution rates (Ks) and nonsynonymous substitution rates (Ka) between each pair of *ParPGRs*. The dotted lines indicate Ka/Ks ratios of 0.1, 0.3, 0.5, and 0.7. The heatmap shows the pairwise Ka/Ks ratios between *ParPGRs*. The figures show the chromosomal position and syntenic relationships of the *PGR* (B), catalase (C) and *CYP337* (D) gene families in three Papilionidae species. The vertical lines represent orthologs among species. (E) Phylogenetic relationships of all the catalase genes.

Figure S9. Functional enrichment of expanded gene families in the ancestor of Papilionidae.

The top ten most significant GO terms and significantly enriched protein families are displayed (hypergeometric test with FDR correction, adjusted *P* < 0.05). The size of the dots indicates the number of genes in the protein family, and the color indicates the significance. The lines connecting protein families overlap with the top function entry.

Figure S10. Rapid evolution of the *CYP3* clan in Papilionidae butterflies.

(A) Bar plots showing the gene numbers of the *CYP6AU1* and *CYP324A1* gene families in each species. (B) Phylogenetic relationships of all the *CYP* genes belonging to the *CYP3* clan in *Pac. aristolochiae*, *S. montelus*, and *Pap. polytes* with *D. melanogaster* as the outgroup. The *CYP337*, *CYP6AU1*, and *CYP324A1* gene families are marked, and the green triangles represent gene copies originating in *Pac. aristolochiae*.

Figure S11. Evolution of *CYP* genes in Papilionidae butterflies.

The phylogenetic relationships of *CYP2* (A), *CYP4* (B), and the mitochondrial *CYP* clans (C) in *Pac. aristolochiae*, *S. montelus*, and *Pap. polytes* with *D. melanogaster* as the outgroup.

Figure S12. Evolution of *GST* in Papilionidae butterflies.

(A) The bar plots show the gene numbers of *GSTD* subfamily in each species. (B) The phylogenetic relationships of all *GST* genes in *Pac. aristolochiae*, *S. montelus*, and *Pap. polytes* with *D. melanogaster* as the outgroup. The *GSTD* subfamily is marked, and the green triangle represents gene copies that originated in *Pac. aristolochiae*.

Figure S13. Gene expression differences between wing disc and body in *Pac. aristolochiae*.

(A) The volcano plot shows the significantly up-regulated (red) and down-regulated (blue) genes in the pupa wing disc compared to the body (Wald test with FDR correction, adjusted *P* < 0.05). (B) Enriched protein families of body up-regulated genes. All significant terms are shown (hypergeometric test with FDR correction, adjusted *P* < 0.05). The size of the dots represents the number of genes and the color represents the significance. (C) Normalized and scaled expression levels of the focal gene families in *Pac. aristolochiae* body and wing disc. (D) The Venn diagram shows the overlaps of differentially expressed genes in exogenous Aristolochic acid metabolism and pupa wing disc development. More genes that responded to exogenous Aristolochic acid are significantly lower expressed in wing discs.

Figure S14. Gene co-expression networks in *Pac. aristolochiae* and *Pap. polytes*.

(A) The principal component analysis results of all samples. The color of the point represents the species and the shape represents the tissue. *Pap. polytes* samples from two stages are included in the analysis. The log-transformed FPKM values are used as the input. (B) The clustering results of all samples. (C) The scale-free topology fit index and mean connectivity are plotted against the soft threshold. An empirical power was used in the subsequent analysis and highlighted in red. (D) The hierarchical clustering tree and the assigned modules of all genes. (E) The heatmap shows the correlations between module eigengene (ME) and the tissue and the species, respectively. Student asymptotic *P* values for given correlations were calculated and * indicates *P* < 0.05, ** indicates *P* < 0.01, and *** indicates *P* < 0.001. B, body. W, wing disc. D6, pupa day 6. D9, pupa day 9.

Figure S15. Function of modules related to tissues and species in *Pac. aristolochiae* and *Pap. polytes*.

(A) Functional enrichment of all genes of module 2. The top five most significant GO terms are displayed (hypergeometric test with FDR correction, adjusted *P* < 0.05). (B) Functional enrichment of hub genes of module 1. The top five most significant GO terms are displayed (hypergeometric test with FDR correction, adjusted *P* < 0.05). (C) KEGG enrichment of module 2 hub genes. All significant KEGG pathways are shown (hypergeometric test, *P* < 0.05). The size of the dots represents the number of genes and the color represents the significance. (D) Normalized and scaled expression levels of module 1 hub genes related to DNA repair and the Fanconi anemia pathway.

Figure S16. Gene co-expression networks in four Papilionidae butterflies.

(A) The clustering results of all samples. The log-transformed FPKM values are used as the input. (B) The scale-free topology fit index and mean connectivity are plotted against the soft threshold. An empirical power was used in the subsequent analysis and highlighted in red. (C) The hierarchical clustering tree and the assigned modules of all genes. (D) The heatmap shows the correlations between module eigengene (ME) and the tissue and species. Student asymptotic *P* values for given correlations were calculated and * indicates *P* < 0.05, ** indicates *P* < 0.01, and *** indicates *P* < 0.001. B, body. W, wing disc. D6, pupa day 6. D9, pupa day 9.

Figure S17. Expression patterns of DNA repair genes in four Papilionidae butterflies.

Normalized and scaled expression levels of module 5 genes related to DNA repair, Fanconi anemia pathway, and nucleotide excision repair pathway. All samples are shown in the heatmap.

Figure S18. NMR spectra of alkyne-tagged AAI.

(A) ^1^H-NMR spectrum (600 MHz) of alkyne-tagged AAI. (B) ^13^C-NMR spectrum (100 MHz) of alkyne-tagged AAI.

Figure S19. Testes isolation from adults of *Pac. aristolochiae*.

(A) Adult. (B) Testis. (C) Administration of monoalkyne AAI to an adult. (D) The embedded testes and thorax tissue after perfusion using OCT.


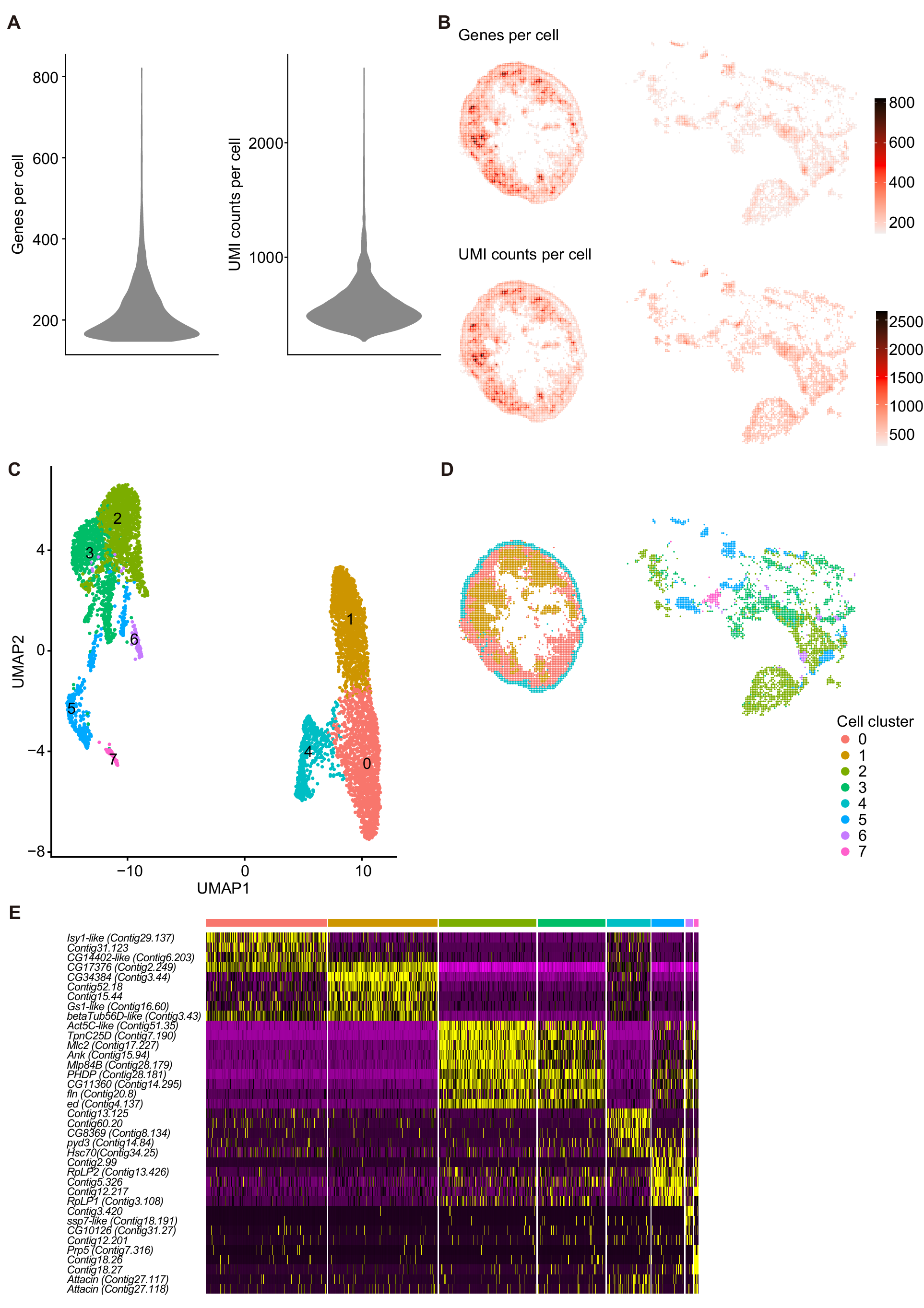


Figure S20. Spatially resolved gene expression patterns in the testis and thorax in *Pac. aristolochiae*.

(A) The violin plots show the number of genes and UMI counts within each bin. (B) The spatial distribution of gene and UMI counts. The darker colors represent higher numbers. (C) The clustering results of all cells in the UMAP space. (D) The spatial distribution of cell clusters. (E) The heatmap shows the expression patterns of the top five marker genes of each cell cluster. Yellow represents higher expression levels.


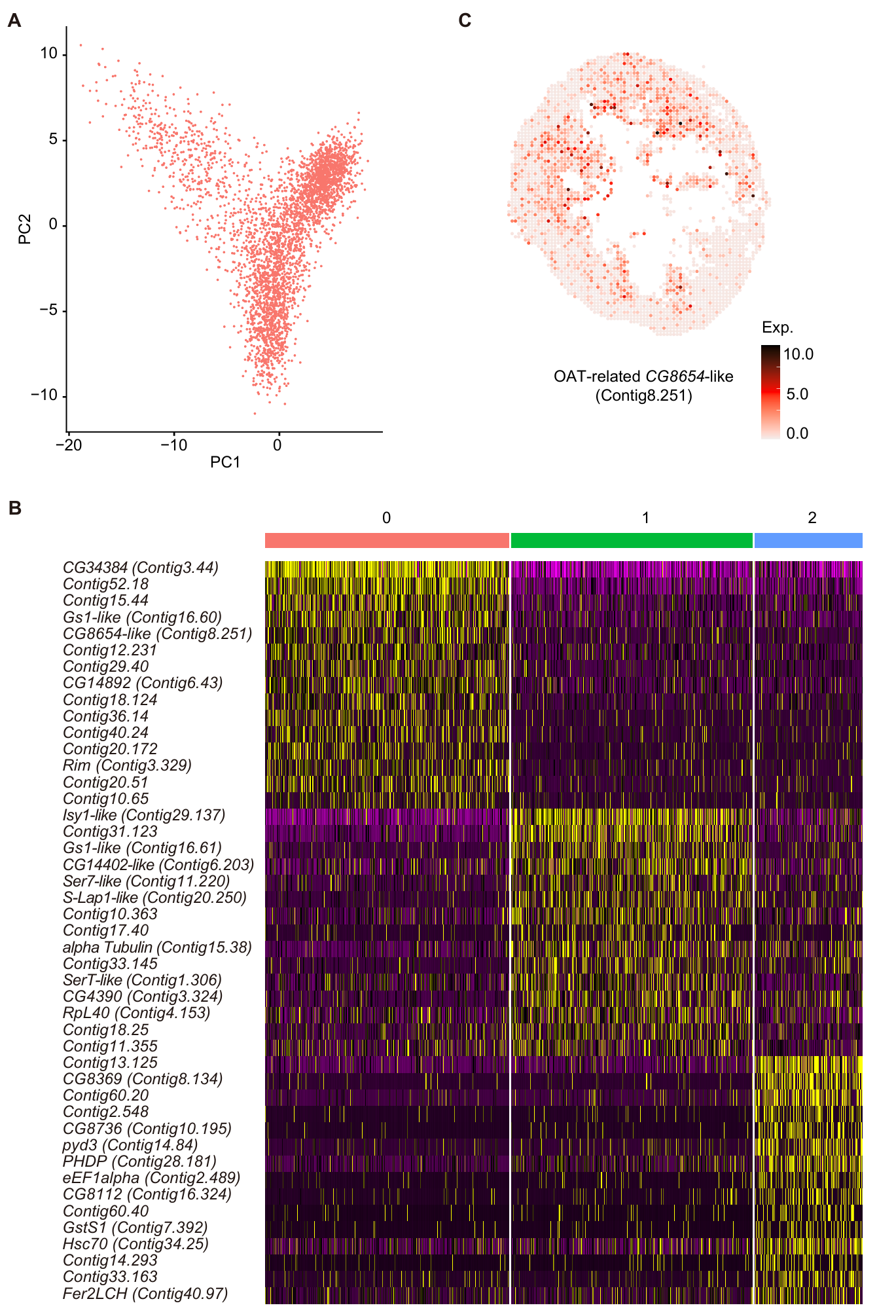


Figure S21. Clustering results of testis cells in *Pac. aristolochiae*.

(A) The principal component analysis results of all cells belonging to the testis. (B) The heatmap shows the expression patterns of the top ten marker genes of each cell cluster. Yellow represents higher expression levels. (C) The spatial expression pattern of the *SLC22* transporter gene *Contig8.251* in the testis.

Figure S22. Evolution of *SLC22* in Papilionidae butterflies.

The phylogenetic relationships of the *SLC22* gene family in *Pac. aristolochiae*, *S. montelus*, and *Pap. polytes* with *D. melanogaster* and *H. sapiens* as the outgroup. OAT, organic anion transporter. OCT, organic cation transporter. OCTN, Organic zwitterions/cation transporter. The green triangle represents the expanded *SLC22* genes in *Pac. aristolochiae*. Although butterflies lack human *OAT* orthologs, a clade containing nine *Pac. aristolochiae SLC22* genes exhibits high sequence similarity to human *OATs* and is defined as OAT-related genes in the figure, including the testes highly expressed gene *Contig8.251*.

**
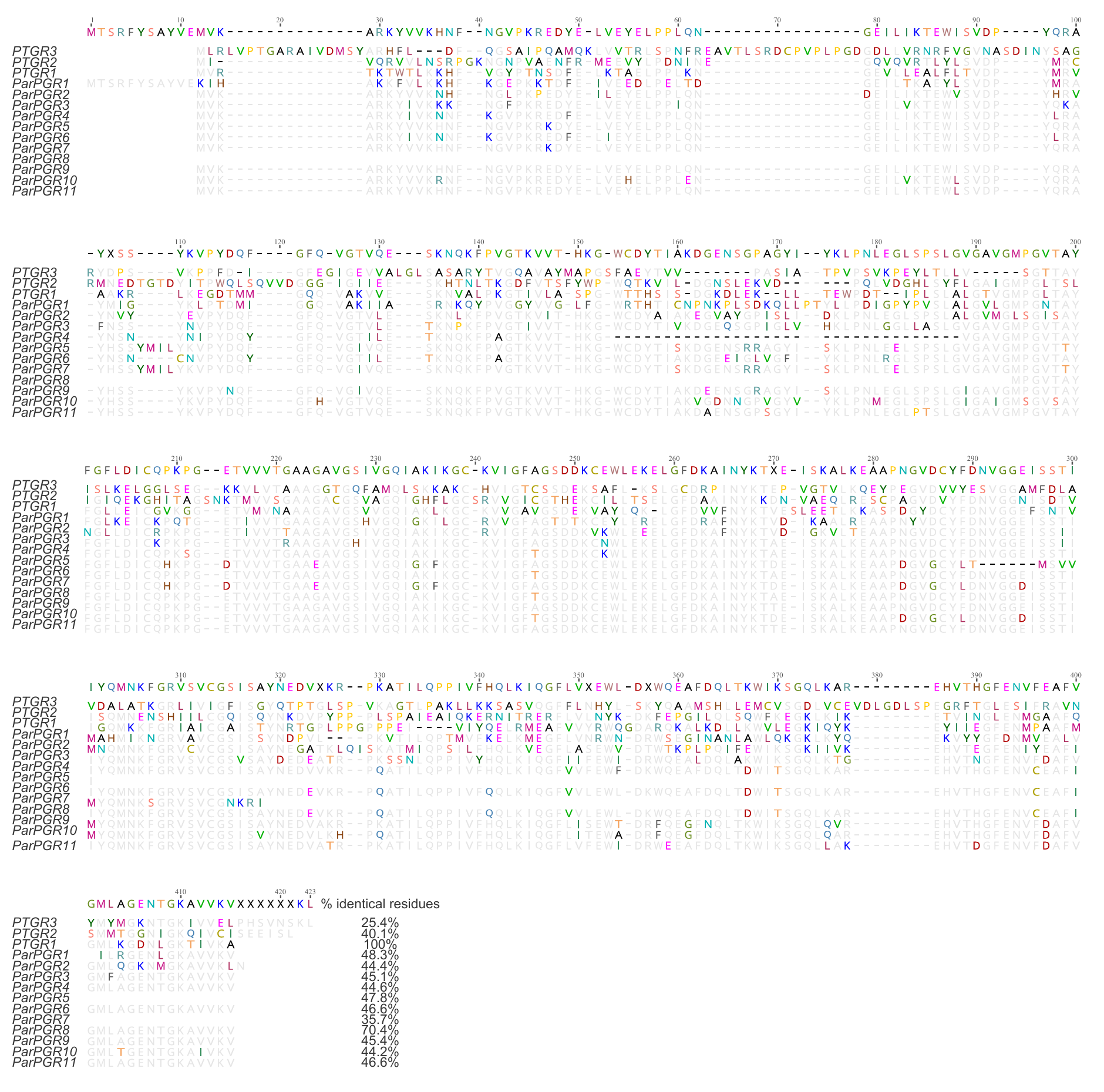
**

Figure S23. Sequence alignments of *ParPGR* genes and human homologs.

The multiple sequence alignment is presented with the consensus sequence shown at the top. Amino acid residues differing from the consensus sequence are highlighted. The pairwise identity of each sequence compared to human *PTGR1* is indicated at the end.

Figure S24. Structure alignments of *ParPGR* genes and human homologs.

(A) The structure of human PTGR1 (PDB accession: 9D6W). The nucleotide-binding domain and catalytic domain are colored in orange and pink, respectively. (B) Structures of human PTGR2 (PDB accession: 2ZB7), and PTGR3 (PDB accession: 7ZEJ). The number of atoms and the root mean square deviation (RMSD) aligned to PTGR1 are shown below the picture. (C) The structures of *Pac. aristolochiae* PGR proteins are predicted using AlphaFold2. The number of atoms and the RMSD aligned to PTGR1 are shown below the picture.

Figure S25. *PTGR1* expression patterns in human kidney single-nucleus atlas.

(A) The UMAP embedding of human kidney single-nucleus atlas with 304,989 nuclei. The color represents the cell cluster. PapE; papillary tip epithelial cell; VSM/P, vascular smooth muscle/pericyte; IMM, immune cell; NEU, neural cell. (B) The distribution of cells derived from healthy reference, AKI and CKD patients. (C) The expression patterns of *PTGR1* in the UMAP reduction. Darker colors represent higher expression levels. (D) The dot plot shows the percentage and average expression level of *PTGR1* across all cell clusters in control and disease conditions. Differential expression analysis between disease groups was performed based on the pseudobulk method (Wald test with FDR correction). The bar plot on the left shows the expression fold change between each cell cluster and the rest of the cells. Only significant and positive expression differences are plotted (Wilcoxon rank sum test with FDR correction). (E) The percentage and average expression level of *PTGR1* in cell subclasses. Only subclasses with significant expression differences between groups are shown (Wald test with FDR correction). Comparison results across cell subclasses are labeled on the left (Wilcoxon rank sum test with FDR correction). (F) Normalized counts of *PTGR1* in reference and disease samples in the specific cell subclass, degenerative proximal tubule epithelial cell (dPT) (Wald test with FDR correction). * indicates adjusted *P* < 0.05, ** indicates adjusted *P* < 0.01 and *** indicates adjusted *P* < 0.001.


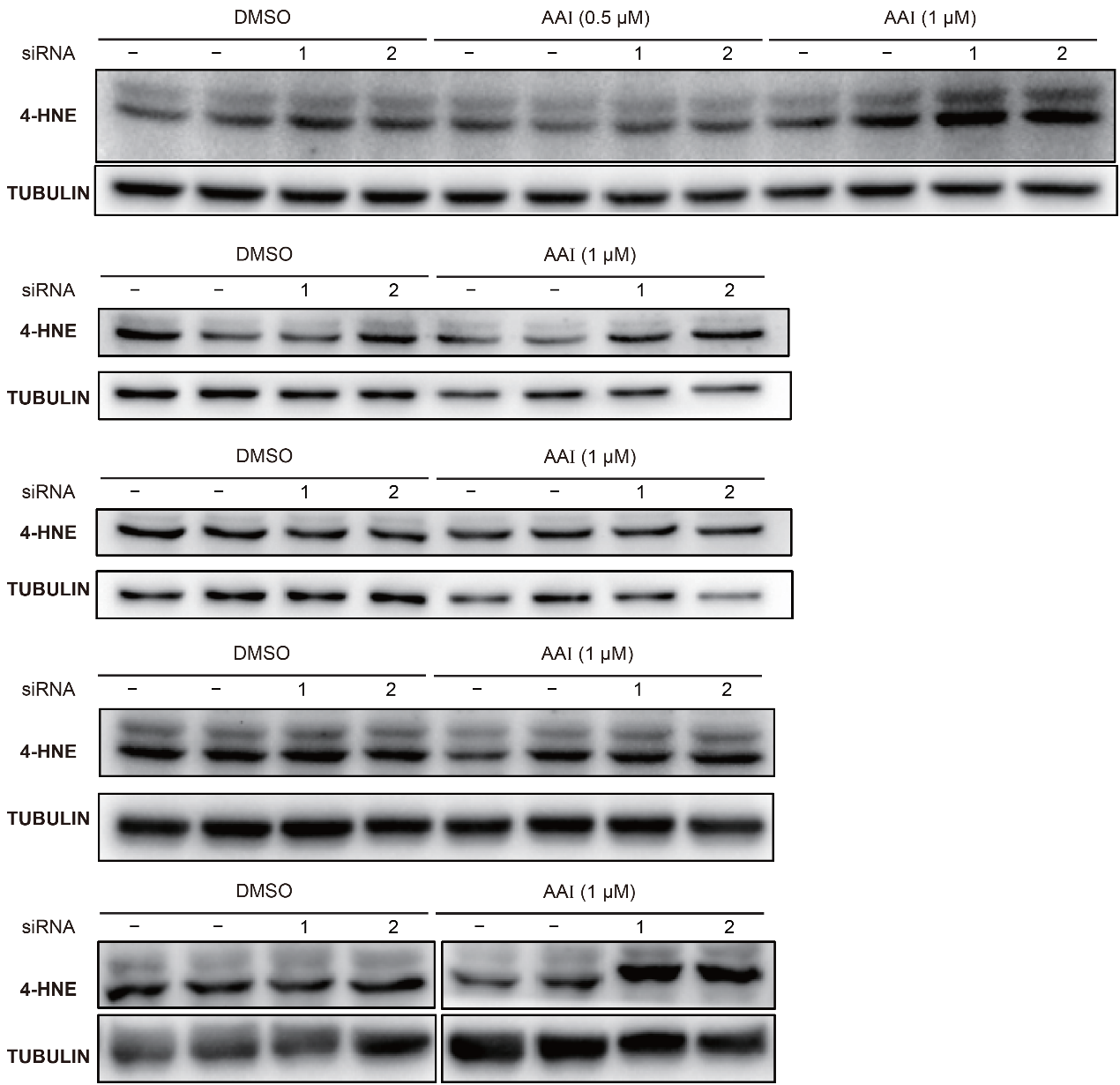


Figure S26. Western blot results of 4-HNE-protein adducts in RT4 cells.

Western blot analyses of 4-HNE and β-tubulin in *PTGR1*-knockdown (siRNA 1 and 2) and negative control cells with and without AAI treatments.

Figure S27. Western blot results of 4-HNE-protein adducts in RT4 cells.

Western blot analyses of 4-HNE, PTGR1, and β-tubulin in *PTGR1* overexpression (OE *PTGR1*) and negative control (EV) cells with and without AAI treatments.

Figure S28. Expression levels of all *PGR* genes in four Papilionidae species.

Data from pupal body samples are used. The same letters represent no significant difference in expression levels, and different letters indicate significant differences (Tukey's HSD test on log-transformed FPKM values, adjusted *P* < 0.05).

Table S1 AAI (16) and possible metabolites (1-15) identified by UHPLC-Q-TOF/MS/MS in the feces of *Pac. aristolochiae* larvae fed on *Aristolochia debilis* leaves coated with AA I solution.

| Peak No.^a^ | t_R_ (min) | Measured*m*/*z* | Possible formula | Calculated *m*/*z* | Error (ppm) | Ion observed | Fragment *m*/*z* ^b^ | Compound identified ^c^ | Intensity ^d^ | Confidence level |
| --- | --- | --- | --- | --- | --- | --- | --- | --- | --- | --- |
| 1 | 7.4 | 310.0329 | C_16_H_7_NO_6_ | 310.0346 | -5.5 | [M+H-H_2_O]^+^ | — | AAIa or isomer | 810 | 3 |
| 2 | 9.1 | 280.0595 | C_16_H_9_NO_4_ | 280.0604 | -3.2 | [M+H]^+^ | — | ALIa or isomer | 696 | 3 |
| 3 | 9.2 | 310.0703 | C_17_H_11_NO_5_ | 310.0710 | -2.3 | [M+H]^+^ | 295.0472 267.0524 | *N-*OH/7-OH-AL I | 4,909 | 2 |
| 4 | 10.1 | 310.0707 | C_17_H_11_NO_5_ | 310.0710 | -1.0 | [M+H]^+^ | 295.0471 267.0535 | *N-*OH/7-OH-AL I | 4,480 | 2 |
| 5 | 10.8 | 310.0712 | C_17_H_11_NO_5_ | 310.0710 | 0.6 | [M+H]^+^ | 295.0455 267.0541 | *N-*OH/7-OH-AL I | 1,868 | 2 |
| 6 | 11.8 | 280.0590 | C_16_H_9_NO_4_ | 280.0604 | -5.0 | [M+H]^+^ | — | ALIa or isomer | 2,002 | 3 |
| 7 | 13.7 | 310.0703 | C_17_H_11_NO_5_ | 310.0710 | -2.3 | [M+H]^+^ | 295.0466 267.0522 | *N-*OH/7-OH-AL I | 4,519 | 2 |
| 8 | 13.8 | 310.0347 | C_16_H_7_NO_6_ | 310.0346 | 0.3 | [M+H-H_2_O]^+^ | 266.0447 238.0501 | AAIa or isomer | 14,233 | 2 |
| 9 | 14.3 | 280.0607 | C_16_H_9_NO_4_ | 280.0604 | 1.1 | [M+H]^+^ | 250.0502 222.0553 | ALIa or isomer | 5,815 | 2 |
| 10 | 15.2 | 310.0365 | C_16_H_7_NO_6_ | 310.0346 | 6.1 | [M+H-H_2_O]^+^ | — | AAIa or isomer | 705 | 3 |
| 11 | 17.0 | 324.0501 | C_17_H_9_NO_6_ | 324.0503 | -0.6 | [M+H-H_2_O]^+^ | 280.0603 265.0379 | AAI isomer | 4,166 | 2 |
| 12 | 17.5 | 324.0507 | C_17_H_9_NO_6_ | 324.0503 | 1.2 | [M+H-H_2_O]^+^ | — | AAI isomer | 1,497 | 3 |
| 13 | 18.5 | 294.0763 | C_17_H_11_NO_4_ | 294.0761 | 0.7 | [M+H]^+^ | — | ALI isomer | 3,422 | 3 |
| 14 | 19.4 | 324.0504 | C_17_H_9_NO_6_ | 324.0503 | 0.3 | [M+H-H_2_O]^+^ | 280.0607 265.0374 | AAI isomer | 57,085 | 2 |
| 15 | 19.7 | 294.0757 | C_17_H_11_NO_4_ | 294.0761 | -1.4 | [M+H]^+^ | 279.0519 251.0574 | ALI | 128,418 | 1 |
| **16** | **20.2** | **324.0501** | **C_17_H_9_NO_6_** | **324.0503** | -0.6 | **[M+H-H_2_O]^+^** | **280.0596 265.0359** | **AAI** | **56,978** | **1** |

(a) Peak numbers are shown in Figure S3.

(b) “—” indicates that no clearly fragmented ions were observed.

(c) The identities of the compounds were determined with reference to the Agilent-Nature Standard Natural Product Personal Compound Database and Library (PCDL) and reported data.

(d) Intensities were normalized against the weights of the samples. Notably, because the intensities shown in Figure S3 represent the total intensities of multiple ions, the values in this table are inconsistent with the intensities of the corresponding chromatographic peaks in Figure S3.

Table S2. Sample information of RNA-seq data.

| Species | Sample ID | Stage/tissue | BioProject | Run | Spots (10^6^) | Bases (Gb) | Alignment rate |
| --- | --- | --- | --- | --- | --- | --- | --- |
| *Pac. aristolochiae* | W1 | Pupa wings | PRJNA1179702 | SRR31156769 | 23.08 | 6.83 | 94.85% |
| *Pac. aristolochiae* | W2 | Pupa wings | PRJNA1179702 | SRR31156768 | 26.71 | 7.91 | 95.03% |
| *Pac. aristolochiae* | W5 | Pupa wings | PRJNA1179702 | SRR31156767 | 26.34 | 7.80 | 94.79% |
| *Pac. aristolochiae* | W7 | Pupa wings | PRJNA1179702 | SRR31156766 | 26.53 | 7.85 | 94.42% |
| *Pac. aristolochiae* | B1 | Pupa abdomen | PRJNA1179702 | SRR31156773 | 30.63 | 9.07 | 88.90% |
| *Pac. aristolochiae* | B2 | Pupa abdomen | PRJNA1179702 | SRR31156772 | 27.75 | 8.22 | 88.69% |
| *Pac. aristolochiae* | B5 | Pupa abdomen | PRJNA1179702 | SRR31156771 | 27.69 | 8.20 | 92.34% |
| *Pac. aristolochiae* | B7 | Pupa abdomen | PRJNA1179702 | SRR31156770 | 26.11 | 7.73 | 92.56% |
| *Pac. aristolochiae* | AA_B1 | Larva body (AAI treatment) | PRJNA665105 | SRR12736785 | 23.60 | 6.99 | 91.94% |
| *Pac. aristolochiae* | AA_B2 | Larva body (AAI treatment) | PRJNA665105 | SRR12736785 | 29.29 | 8.67 | 84.45% |
| *Pac. aristolochiae* | AA_B3 | Larva body (AAI treatment) | PRJNA665105 | SRR12736785 | 25.75 | 7.62 | 80.53% |
| *Pac. aristolochiae* | Control_B1 | Larva body (control) | PRJNA665105 | SRR12736787 | 31.58 | 9.35 | 90.35% |
| *Pac. aristolochiae* | Control_B2 | Larva body (control) | PRJNA665105 | SRR12736787 | 31.78 | 9.41 | 88.71% |
| *Pac. aristolochiae* | Control_B4 | Larva body (control) | PRJNA665105 | SRR12736787 | 24.27 | 7.18 | 90.01% |
| *Pap. polytes* | P6F1 | Pupa forewings (Day 6, female) | PRJNA634605 | SRR11834790 | 15.10 | 2.99 | 94.05% |
| *Pap. polytes* | P6F2 | Pupa forewings (Day 6, female) | PRJNA634605 | SRR11834785 | 11.28 | 2.27 | 92.81% |
| *Pap. polytes* | P6F3 | Pupa forewings (Day 6, male) | PRJNA634605 | SRR11834779 | 14.05 | 2.81 | 94.71% |
| *Pap. polytes* | P6F4 | Pupa forewings (Day 6, male) | PRJNA634605 | SRR11834774 | 16.36 | 3.24 | 94.54% |
| *Pap. polytes* | P6H1 | Pupa hindwings (Day 6, female) | PRJNA634605 | SRR11834789 | 13.80 | 2.73 | 93.18% |
| *Pap. polytes* | P6H2 | Pupa hindwings (Day 6, female) | PRJNA634605 | SRR11834784 | 9.92 | 1.99 | 93.85% |
| *Pap. polytes* | P6H3 | Pupa hindwings (Day 6, male) | PRJNA634605 | SRR11834778 | 14.51 | 2.90 | 94.63% |
| *Pap. polytes* | P6H4 | Pupa hindwings (Day 6, male) | PRJNA634605 | SRR11834773 | 14.65 | 2.90 | 91.94% |
| *Pap. polytes* | P6B1 | Pupa abdomen (Day 6, female) | PRJNA634605 | SRR11834786 | 14.14 | 2.80 | 92.98% |
| *Pap. polytes* | P6B2 | Pupa abdomen (Day 6, female) | PRJNA634605 | SRR11834780 | 9.75 | 1.96 | 77.69% |
| *Pap. polytes* | P6B3 | Pupa abdomen (Day 6, male) | PRJNA634605 | SRR11834775 | 13.78 | 2.73 | 93.76% |
| *Pap. polytes* | P6B4 | Pupa abdomen (Day 6, male) | PRJNA634605 | SRR11834769 | 16.79 | 3.32 | 93.72% |
| *Pap. polytes* | P9F1 | Pupa forewings (Day 9, female) | PRJNA634605 | SRR11834768 | 15.59 | 3.07 | 92.61% |
| *Pap. polytes* | P9F2 | Pupa forewings (Day 9, female) | PRJNA634605 | SRR11834763 | 15.38 | 3.05 | 96.14% |
| *Pap. polytes* | P9F3 | Pupa forewings (Day 9, male) | PRJNA634605 | SRR11834757 | 16.87 | 3.32 | 95.04% |
| *Pap. polytes* | P9F4 | Pupa forewings (Day 9, male) | PRJNA634605 | SRR11834752 | 17.02 | 3.37 | 94.68% |
| *Pap. polytes* | P9H1 | Pupa hindwings (Day 9, female) | PRJNA634605 | SRR11834767 | 17.30 | 3.42 | 95.61% |
| *Pap. polytes* | P9H2 | Pupa hindwings (Day 9, female) | PRJNA634605 | SRR11834762 | 15.91 | 3.15 | 95.99% |
| *Pap. polytes* | P9H3 | Pupa hindwings (Day 9, male) | PRJNA634605 | SRR11834756 | 16.47 | 3.26 | 95.24% |
| *Pap. polytes* | P9H4 | Pupa hindwings (Day 9, male) | PRJNA634605 | SRR11834751 | 16.00 | 3.17 | 95.26% |
| *Pap. polytes* | P9B1 | Pupa abdomen (Day 9, female) | PRJNA634605 | SRR11834764 | 16.29 | 3.22 | 92.12% |
| *Pap. polytes* | P9B2 | Pupa abdomen (Day 9, female) | PRJNA634605 | SRR11834758 | 16.23 | 3.20 | 93.60% |
| *Pap. polytes* | P9B3 | Pupa abdomen (Day 9, male) | PRJNA634605 | SRR11834753 | 9.82 | 1.94 | 91.50% |
| *Pap. polytes* | P9B4 | Pupa abdomen (Day 9, male) | PRJNA634605 | SRR11834863 | 10.14 | 2.04 | 89.12% |
| *Pap. xuthus* | PX1 | Pupa abdomen | PRJNA718996 | SRR14141645 | 22.69 | 6.72 | 94.10% |
| *Pap. xuthus* | PX2 | Pupa abdomen | PRJNA718996 | SRR14141653 | 23.60 | 6.96 | 92.60% |
| *Pap. xuthus* | PX3 | Pupa abdomen | PRJNA718996 | SRR14141654 | 29.70 | 8.79 | 93.72% |
| *Pap. xuthus* | PX4 | Pupa abdomen | PRJNA270384 | SRR1760414 | 26.56 | 4.62 | 89.41% |
| *Pap. xuthus* | PX5 | Pupa abdomen | PRJNA270384 | SRR1760413 | 24.94 | 4.34 | 91.54% |
| *Pap.machaon* | PM1 | Pupa abdomen | PRJNA728224 | SRR14478593 | 23.08 | 6.83 | 84.39% |
| *Pap.machaon* | PM2 | Pupa abdomen | PRJNA728224 | SRR14478601 | 28.63 | 8.47 | 84.56% |
| *Pap.machaon* | PM3 | Pupa abdomen | PRJNA728224 | SRR14478602 | 20.84 | 6.15 | 82.50% |
| *Pap.machaon* | PM4 | Pupa abdomen | PRJNA270386 | SRR1760420 | 31.47 | 5.44 | 89.22% |
| *Pap.machaon* | PM5 | Pupa abdomen | PRJNA270386 | SRR1760419 | 26.36 | 4.59 | 89.91% |

Table S3. Best hit of candidate genes in reciprocal BLAST between *Pac. aristolochiae* and human.

| *Pac. aristolochiae* to GRCh38 | | | | GRCh38 to *Pac. aristolochiae* | | | |
| --- | --- | --- | --- | --- | --- | --- | --- |
| Query ID | Subject ID | Identity percentage | E value | Query ID | Subject ID | Identity percentage | E value |
| ParPGR1(Contig1.79) | PTGR1 | 47.16 | 8.00E-100 | PTGR1 | ParPGR11(Contig1.747) | 47.04 | 2.00E-89 |
| ParPGR2(Contig1.653) | PTGR1 | 44.08 | 7.00E-96 | PTGR1 | ParPGR11(Contig1.747) | 47.04 | 2.00E-89 |
| ParPGR11(Contig1.747) | PTGR1 | 47.04 | 4.00E-87 | PTGR1 | ParPGR11(Contig1.747) | 47.04 | 2.00E-89 |
| ParPGR4(Contig1.741) | PTGR1 | 44.11 | 5.00E-80 | PTGR1 | ParPGR11(Contig1.747) | 47.04 | 2.00E-89 |
| Contig8.365 | SLC22A3 | 28.08 | 1.00E-66 | SLC22A3 | Contig8.249 | 38.71 | 3.00E-79 |
| Contig24.256 | LIPF | 35.33 | 5.00E-66 | LIPF | Contig5.411 | 40.05 | 2.00E-94 |
| Contig8.367 | SLC22A1 | 31.91 | 1.00E-63 | SLC22A1 | Contig8.249 | 33.41 | 2.00E-70 |
| Contig15.71 | SLC22A3 | 29.93 | 1.00E-62 | SLC22A3 | Contig8.249 | 38.71 | 3.00E-79 |
| Contig24.250 | LIPF | 33.07 | 2.00E-62 | LIPF | Contig5.411 | 40.05 | 2.00E-94 |
| Contig24.246_2 | LIPK | 32.29 | 3.00E-48 | LIPK | Contig5.411 | 35.66 | 7.00E-86 |
| Contig3.37 | CYP3A7 | 29.89 | 3.00E-46 | CYP3A7 | Contig30.19 | 32.54 | 4.00E-84 |
| Contig1.29 | CYP3A5 | 28.71 | 7.00E-46 | CYP3A5 | Contig30.19 | 34.00 | 3.00E-74 |
| Contig3.36 | CYP3A7 | 29.65 | 2.00E-40 | CYP3A7 | Contig30.19 | 32.54 | 4.00E-84 |
| Contig10.139 | ACSF2 | 26.13 | 5.00E-39 | ACSF2 | Contig4.69 | 38.98 | 2.00E-142 |
| Contig3.34 | CYP3A7 | 29.80 | 2.00E-38 | CYP3A7 | Contig30.19 | 32.54 | 4.00E-84 |
| Contig3.33_2 | CYP3A7 | 28.61 | 3.00E-37 | CYP3A7 | Contig30.19 | 32.54 | 4.00E-84 |
| Contig3.35 | CYP3A5 | 27.73 | 5.00E-37 | CYP3A5 | Contig30.19 | 34.00 | 3.00E-74 |
| Contig10.138 | ACSM4 | 25.66 | 7.00E-34 | ACSM4 | Contig20.35 | 27.85 | 8.00E-42 |
| Contig12.335 | FDPS | 26.67 | 2.00E-33 | FDPS | Contig12.270 | 42.27 | 7.00E-96 |
| Contig2.336 | ACSF2 | 29.83 | 5.00E-33 | ACSF2 | Contig4.69 | 38.98 | 2.00E-142 |
| Contig10.136 | ACSF2 | 29.32 | 1.00E-27 | ACSF2 | Contig4.69 | 38.98 | 2.00E-142 |
| Contig10.134 | ACSF2 | 24.32 | 1.00E-25 | ACSF2 | Contig4.69 | 38.98 | 2.00E-142 |
